# Biophysical Fitness Landscapes for Transcription Factor Binding Sites

**DOI:** 10.1371/journal.pcbi.1003683

**Published:** 2014-07-10

**Authors:** Allan Haldane, Michael Manhart, Alexandre V. Morozov

**Affiliations:** 1 Department of Physics and Astronomy, Rutgers University, Piscataway, New Jersey, United States of America; 2 BioMaPS Institute for Quantitative Biology, Rutgers University, Piscataway, New Jersey, United States of America; University of California, San Diego, United States of America

## Abstract

Phenotypic states and evolutionary trajectories available to cell populations are ultimately dictated by complex interactions among DNA, RNA, proteins, and other molecular species. Here we study how evolution of gene regulation in a single-cell eukaryote *S. cerevisiae* is affected by interactions between transcription factors (TFs) and their cognate DNA sites. Our study is informed by a comprehensive collection of genomic binding sites and high-throughput *in vitro* measurements of TF-DNA binding interactions. Using an evolutionary model for monomorphic populations evolving on a fitness landscape, we infer fitness as a function of TF-DNA binding to show that the shape of the inferred fitness functions is in broad agreement with a simple functional form inspired by a thermodynamic model of two-state TF-DNA binding. However, the effective parameters of the model are not always consistent with physical values, indicating selection pressures beyond the biophysical constraints imposed by TF-DNA interactions. We find little statistical support for the fitness landscape in which each position in the binding site evolves independently, indicating that epistasis is common in the evolution of gene regulation. Finally, by correlating TF-DNA binding energies with biological properties of the sites or the genes they regulate, we are able to rule out several scenarios of site-specific selection, under which binding sites of the same TF would experience different selection pressures depending on their position in the genome. These findings support the existence of universal fitness landscapes which shape evolution of all sites for a given TF, and whose properties are determined in part by the physics of protein-DNA interactions.

## Author Summary

Specialized proteins called transcription factors turn genes on and off by binding to short stretches of DNA in their regulatory regions. Precise gene regulation is essential for cellular survival and proliferation, and its evolution and maintenance under mutational pressure are central issues in biology. Here we discuss how evolution of gene regulation is shaped by the need to maintain favorable binding energies between transcription factors and their genomic binding sites. We show that, surprisingly, transcription factor binding is not affected by many biological properties, such as the essentiality of the gene it regulates. Rather, all sites for a given factor appear to evolve under a universal set of constraints, which can be rationalized in terms of a simple model inspired by transcription factor – DNA binding thermodynamics.

## Introduction

A powerful concept in evolution is the fitness landscape: for every possible genotype there is a number, known as the genotypic fitness, that characterizes the evolutionary success of that genotype [1]. Evolutionary success is typically quantified as the probability of surviving to reproduce, number of offspring, growth rate, or a related proxy [2], [3]. The structure of the fitness landscape is key to understanding the evolutionary fates of populations.

Most traditional studies of molecular evolution rely on simplified models of fitness landscapes [3]–[6] or empirical reconstructions of landscapes based on limited experimental data [3], [7]–[10]. However, fitness landscapes are fundamentally shaped by an intricate network of interactions involving DNA, RNA, proteins, and other molecular species present in the cell. Thus we should be able to cast these landscapes in terms of biophysical properties such as binding affinities, molecular stabilites, and degradation rates. The increasing availability of quantitative high-throughput data on *in vitro* and *in vivo* molecular interactions has led to growing efforts aimed at developing models of evolution that explicitly incorporate the underlying biophysics [11]–[25]. These models combine evolutionary theory with physical models of molecular systems, for example focusing on how protein folding stability or specificity of intermolecular interactions shapes the ensemble of accessible evolutionary pathways and steady-state distributions of biophysical phenotypes.

Evolution of gene regulation is particularly well-suited to this type of analysis. Gene activation and repression are mediated by binding of transcription factors (TFs) to their cognate genomic sites. These binding sites are short nucleotide sequences, typically 5–25 bp in length, in gene promoters that interact specifically with TF DNA-binding domains [26]. In eukaryotes, a given TF can have numerous binding sites in the genome, and many genes are regulated by several TFs [26], [27]. Understanding TF-mediated regulation is key to understanding complex regulatory networks within eukaryotic cells − one of the main challenges facing molecular biology. Moreover, the availability of high-throughput data on the genomic locations of TF binding sites [28]–[31] and on TF-DNA energetics [32]–[35] make it possible to develop biophysical models of evolution of gene regulation.

Here we consider evolution of TF binding sites in the yeast *Saccharomyces cerevisiae*. We study how energetics of protein-DNA interactions affect the structure of the binding site fitness landscape. In a significant extension of previous work which analyzed a single yeast TF [22], we consider a collection of 25 *S. cerevisiae* TFs for which models of TF binding energetics were built using high-throughput *in vitro* measurements of TF-DNA interactions [35]. We focus on 12 TFs for which sufficient data on genomic sites [31] are also available. We use a model of monomorphic populations undergoing consecutive substitutions [19], [36]–[38] to infer fitness landscapes, as functions of TF-DNA binding energy, from observed distributions of TF binding sites in the yeast genome [22]. In contrast to the previous work [22], we rationalize these fitness landscapes in terms of a simple parametric model based on thermodynamics of TF-DNA binding, obtaining explicit values of effective evolutionary parameters. Our analysis sheds light on the genome-wide importance of TF-DNA interactions in regulatory site evolution.

Moreover, we investigate the hypothesis that universal biophysical constraints, rather than site-specific selective pressures, dominate evolution of regulatory sites. We test the relationship between TF binding energies and various biological properties, such as the essentiality of the corresponding gene [39]. We find no clear relationship between physical and biological properties of TF sites, which indicates that evolution of site energetics is largely insensitive to site-specific biological functions and is therefore driven by global biophysical constraints.

## Results

### 1 Biophysical model of TF binding site evolution

#### 1.1 Energetics of TF-DNA binding

The probability of a binding site to be bound by a TF is given by the Fermi-Dirac function of the free energy 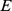 of TF-DNA interaction [40]:
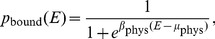
(1)where 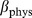 is the physical inverse temperature (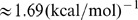 at room temperature), and 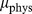 is the physical chemical potential, a function of the TF concentration. The binding energy 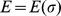 of a site is a function of its nucleotide sequence, 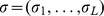, where 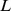 is the length of the site and 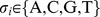. Note that 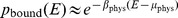 if 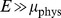. In the mean-field approximation, each nucleotide makes an additive contribution to the total energy of the site [32]. These contributions are parameterized by an energy matrix, whose entries 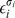 give the contribution to the total energy from the nucleotide 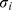 at position 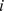:
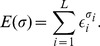
(2)Energy matrices can be readily generalized to more complex models of sequence-dependent energetics, such as those with contributions from dinucleotides, although here for simplicity we focus on the additive model.

#### 1.2 Evolutionary model

We consider a population with a locus in the monomorphic limit: mutations in the locus are infrequent enough that each new mutation either fixes or goes extinct before a second mutation arises [36]. This approximation is valid in the limit 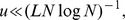 where 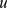 is the mutation rate (probability of mutation per base per generation), 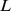 is the number of bases in the locus, and 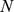 is an effective population size [41]. Indeed, in the monomorphic limit the expected time between new mutations, 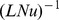, must be longer than the expected time over which fixation occurs, which is 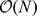 generations with probability 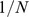 for mutants that fix, and 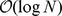 with probability 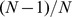 for mutants that go extinct. Thus the total expected time before the mutant either fixes or goes extinct is 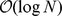 generations for 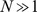 [42]. Thus we must have 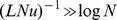 or, equivalently, 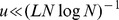. We also assume that the locus is unlinked to the rest of the genome by recombination, and thus we can consider its evolution independently. In evolutionary steady state, the probability that the population has genotype 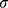 at the locus is given by [19], [37], [38]

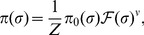
(3)where 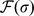 is the multiplicative fitness (defined so that the total fitness of a set of independently evolving loci is a product of fitnesses for each one), 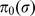 is the neutral distribution of sequences (steady state under no selection), and 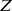 is a normalization constant. The exponent 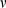 is a “scaling” effective population size which is closely related to the standard variance effective population size [38]. For example, 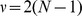 in the Wright-Fisher model and 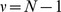 in the Moran model of population genetics [43]. Conceptually, both 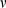 and 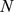 measure the strength of genetic drift [36].

The distribution in Eq. 3 is valid for a wide class of population models [38] (see Methods for details). An analogy with statistical mechanics is suggested by rewriting Eq. 3 as a Boltzmann distribution [37]:
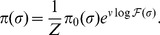
(4)Here the logarithm of fitness plays the role of a negative Hamiltonian, and the neutral distribution 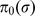 plays the role of entropy. Typically we expect relatively few sequences with high fitness and many with low fitness; thus mutations will drive the population toward lower fitness, while selection will favor higher fitness. The balance between these two competing forces depends on the effective population size 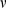, which controls the strength of random fluctuations and is analogous to inverse temperature in the Boltzmann distribution.

#### 1.3 Biophysical model of binding site evolution

Since we are primarily interested in the biophysical aspects of binding site evolution, it is more convenient to consider evolution in the space of binding energies by projecting Eq. 3 via the sequence-energy mapping of Eq. 2:
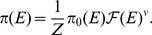
(5)Here the binding site fitness 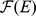 depends only on the binding energy 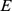. As a general ansatz, we will assume that the fitness depends on the binding energy through the physical binding probability 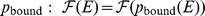. Further, we assume that an organism with an always-bound site (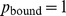, 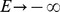) has fitness 1, while an organism with a site that never binds (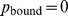, 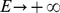) has fitness 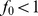. Since real sites are somewhere in between these extremes, a simple hypothesis for the fitness function is an average of these two fitness values weighted by the thermodynamic probabilities of the site being bound or unbound:
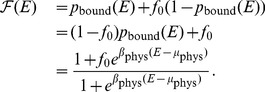
(6)
Equation 6 assumes that the fitness function depends linearly on the TF binding probability 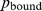, which equals the site's average occupancy. However, this linear dependence may be too restrictive. For example, it does not account for the scenario in which a cell only requires 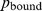 to be above some minimum threshold 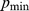, such that the fitness equals 1 when 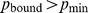, and 0 otherwise. To include a wider range of fitness functions, we extend our model in Eq. 6 by treating 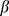 and 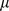 as effective fitting parameters (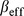 and 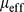) that may deviate from their physical counterparts:
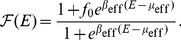
(7)When 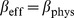 and 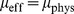, Eq. 7 is equivalent to Eq. 6 and fitness is linearly proportional to 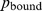, but deviations between these effective and physical parameters introduce nonlinear dependence of fitness on 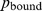. For example, the case in which 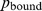 must only exceed a minimum threshold 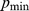 is equivalent to Eq. 7 with 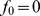, 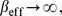 and 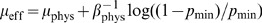. For the remainder of the paper, we will focus on the effective fitness function of Eq. 7 and infer its parameters from data. Thus for simplicity we will drop the explicit “eff” labels on 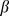 and 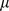.

An important feature of Eq. 3 is that we may invert it to obtain the fitness function in terms of the observed steady-state distributions 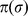 and 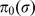, or 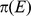 and 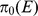 in energy space [19]:
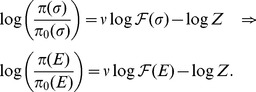
(8)Thus, given a distribution of evolved binding site sequences 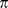 and a neutral distribution 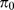, we can use Eq. 8 to infer the logarithm of the fitness landscape up to an overall scale and shift. This can be done without any *a priori* knowledge of the shape of the fitness function. Moreover, given a specific functional form of 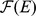, such as the effective Fermi-Dirac fitness in Eq. 7, we can perform a maximum likelihood fit of the observed sequence distribution to infer values of parameters 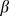, 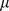, 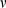, and 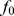. The resulting fitted function can be evaluated by comparison to the general inference in Eq. 8.

When 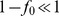, 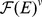 contains an approximate degeneracy in terms of 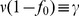, i.e., all fitness functions with constant 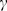 are approximately equivalent. Indeed, the steady-state distribution in Eq. 5 depends on the quantity 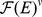, which can be written as
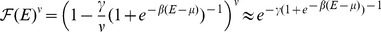
(9)if 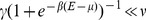 or, since 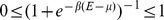, if 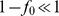. Therefore in this limit, the steady-state distribution 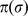 depends only on the parameter 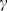 and not on 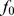 and 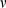 separately.

This degeneracy in the steady-state distribution is not surprising in light of the underlying population genetics, which also provides an interpretation of 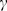. The quantity 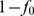 is the selection coefficient 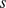 between the two phenotypes of the system, e.g., the bound and unbound states of the TF binding site. As discussed above, the quantity 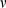 is an effective population size, which sets the strength 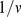 of genetic drift. When 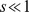 and 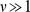, steady-state properties of the population (e.g., allele frequency distribution, fixation probability) are described by the diffusion limit and mathematically depend only on 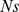, or in our model, 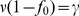 [43], [44], which quantifies the strength of selection relative to the strength of drift. When 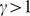, selection is strong relative to drift, while 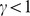 indicates that selection is relatively weak. Note that only the absolute magnitude of the selection coefficient 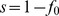 is required to be small for this degeneracy to hold; the selection strength relative to drift, quantified by 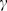, may still be large.

Two regions of parameter space also exhibit a degeneracy between 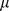 and 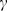. If 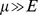 for all site energies 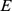, all of the observed sites are predicted to be highly occupied and 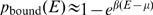. We may thus approximate
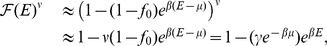
(10)and thus all fitness functions with constant 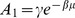 are approximately equivalent. One can thus make 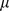 arbitrarily large (while holding 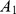 fixed by varying 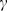) without breaking the degeneracy. If 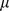 is decreased the degeneracy will eventually break as 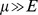 is violated. A similar degeneracy appears when 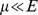, as then 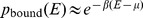; if additionally 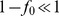, then
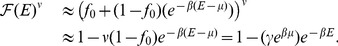
(11)(We can remove an overall factor of 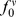 because the distribution 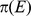 in Eq. 5 is invariant under an overall rescaling of fitness.) Therefore all fitness functions with 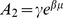 are approximately equivalent in this case. Here, 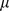 can be made arbitrarily negative without breaking the degeneracy.

Thus, parameter fits fall into three cases for different TFs: If 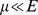, TF-DNA binding energies fit to the right (exponential) end of the Fermi-Dirac function, and we cannot infer a unique 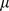. Similarly, if 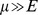, TF-DNA binding energies fit to the left (high occupancy) side of the Fermi-Dirac function, and we again cannot infer 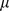 precisely. However, if 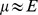, neither degeneracy holds and a unique 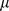 can be inferred. Despite the fact that 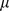 cannot always be predicted, we can unambiguously classify each fit into one of these three cases.

#### 1.4 Selection strength and its dependence on biophysical parameters

We now consider how changes to biophysical parameters of the model affect the strength of selection on binding sites. The selection coefficient for a mutation with small change in energy 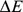 is
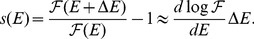
(12)Therefore we can characterize local variations in the strength of selection by considering 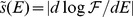, the per-unit-energy local selection coefficient. For the Fermi-Dirac landscape, we obtain
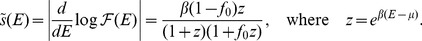
(13)We use the absolute value here since the sign of the selection coefficient is always unambiguous, as the Fermi-Dirac function decreases monotonically with energy.

**Figure 1:**
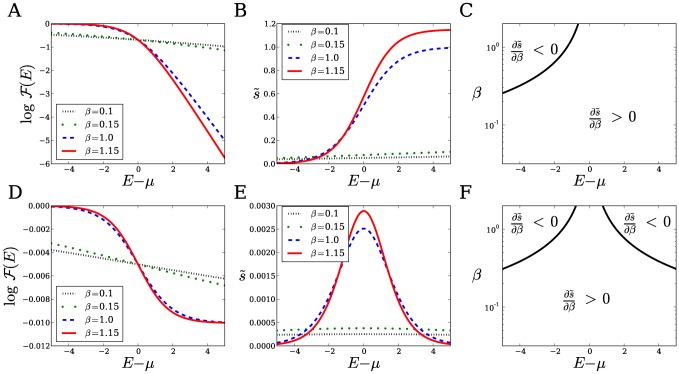
Fitness and selection strength as functions of energy and inverse temperature. Energy 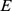 is measured with respect to the chemical potential 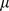. Top row uses 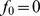; bottom row uses 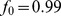. (A,D) Logarithm of Fermi-Dirac fitness versus energy for several values of 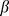; note that the high-energy tail looks distinctly different when 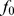 is nonzero. (B,E) Per-unit-energy selection strength 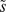 versus energy for several values of 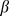; note that the relative ordering of selection strength curves depends on the value of 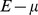. (C,F) Sign of derivative of selection strength with respect to 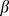, as a function of 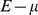 and 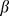. Black boundary in (C) is the curve 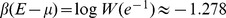, where 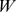 is the Lambert W-function; the boundaries in (F) are the curves 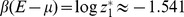 and 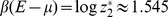 where 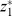, 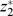 are the solutions to 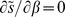 (Eq. 14) with 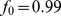.

We can also ask how variations in 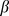 affect the local strength of selection. Variation of 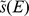 with 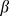 depends qualitatively on both 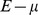 and whether 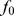 is zero or nonzero. In Fig. 1 we show 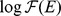, 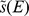, and the derivative
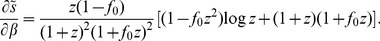
(14)For 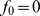 (Fig. 1A–C), increasing 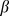 increases selection strength for 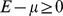. Here the fitness function drops to zero exponentially, and increasing 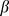 steepens the exponential drop. However, for 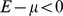, the effect of changing 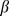 depends on the value of 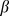 relative to 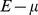. For large 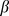, increasing 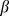 actually decreases selection strength; this is because 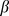 sets the rate at which the Fermi-Dirac function converges to unity, and hence increasing 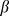 flattens the landscape in that region. However, for sufficiently small 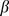, the threshold region is large enough that increasing 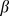 still increases selection. The boundary between positive and negative values of 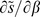 are the solutions of the equation 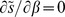: 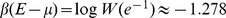, where 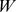 is the Lambert W-function (Fig. 1C).

This situation changes qualitatively in the regime 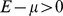 when 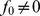 (Fig. 1D–F). In this case, for sufficiently large 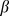, increasing 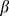 weakens selection. This is different in the case of nonzero 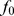 because on the high-energy tail, the fitness is converging to a nonzero number 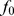, and thus selection becomes asymptotically neutral. Hence, when 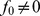, increasing 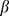 only strengthens selection very close to 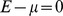. Using Eq. 14, the boundaries in Fig. 1F are given by the solutions of 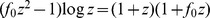. This equation can be solved numerically to obtain two solutions, 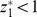 and 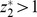. The boundaries in Fig. 1F are thus given by the curves 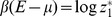 for 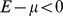 and 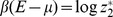 for 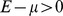.

#### 1.5 Assessment of model assumptions

Two main assumptions inherent in our evolutionary model are monomorphism and steady state. Here, we assess how violating these assumptions affects inference of evolutionary parameters 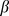, 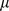, 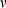, and 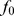. To test this, we generate simulated data sets of binding site sequences evolving under a haploid asexual Wright-Fisher model with the Fermi-Dirac fitness function (Eq. 7; see Methods for details).

**Figure 2:**
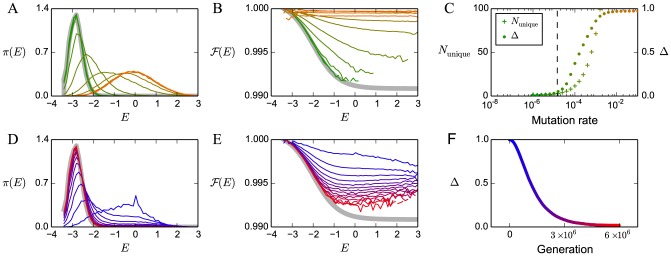
The monomorphic limit and steady state of a Wright-Fisher model of population genetics. In (A)–(C) we show results from simulations at various mutation rates, using a fitness function with 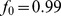, 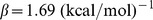, and 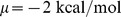. Each mutation rate data point is an average over 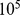 independent runs, as described in Methods. Colors from green to orange correspond to increasing mutation rates. (A) Observed steady-state distributions 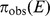 for various mutation rates. The steady state 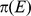 predicted using Eq. 3 is shown in gray. (B) Fitness functions 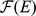 predicted using observed distributions 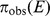 in Eq. 8. The exact fitness function is shown in gray. Inferred fitness functions are matched to the exact one by using the known population size 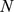, and setting the maximum fitness to 1.0 for each curve. (C) For each mutation rate, the total variation distance (TVD) 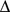 between 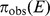 and 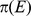, and the average number of unique sequences in the population 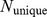 (the degree of polymorphism) are shown. The predicted bound 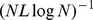 on mutation rate required for monomorphism is shown as a dashed line. In (D)–(F) we show simulations in the monomorphic regime which have not reached steady state, with the same parameters as in (A)–(C) and 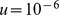. Colors from blue to red correspond to the increasing number of generations.

*Deviations from the monomorphic limit*. To test the effects of polymorphism on the accuracy of our predictions, we perform a set of simulations for a range of mutation rates 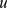. Each simulation in the set follows the Wright-Fisher process to the steady state. We construct the observed distribution 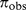 by randomly choosing a single sequence from the final population of each simulation, which may not be monomorphic for larger 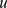 (Fig. 2A). From 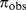, we carry out maximum-likelihood inference of the fitness landscape as a function of energy using Eq. 5 (Fig. 2B), as described in Methods.

Additionally, for each 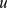 we record the average number of unique sequences present in the population in steady state and compute the total variation distance (TVD; Eq. 25 in Methods) between 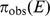 and the monomorphic prediction 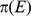 using Eq. 5 (Fig. 2C). The TVD ranges from zero for identical distributions to unity for completely non-overlapping dis tributions. As expected, at low mutation rates the steady-state distribution and the fitness function match monomorphic predictions well. At higher mutation rates, the TVD starts to increase and Eq. 3 overestimates the fitness of low-affinity sites. The population becomes polymorphic in this limit. With very high mutation rates, 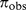 approaches the neutral distribution 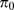 since the population is largely composed of newly generated mutants which have not experienced selection. A condition for monomorphism in a neutrally evolving population is 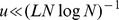 [41]. Using 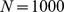 and 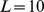 as in our simulations yields 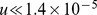 in the monomorphic limit, consistent with the results in Fig. 2C.

**Figure 3:**
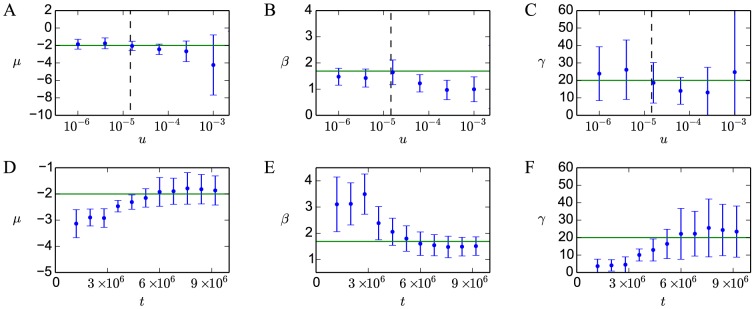
Fitted parameters of the Fermi-Dirac function from Wright-Fisher simulations. In (A)–(C) the fitted values of 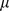, 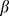 and 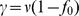 are shown as functions of mutation rate 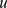. For each mutation rate, we generate 200 random samples of 500 sequences from the 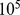 sequences generated in simulations used in Fig. 2A–C. We fit the parameters of the fitness function on each sample separately by maximum likelihood (see Methods). Shown are the averages (points) and standard deviations (error bars) over 200 samples at each mutation rate. The exact values used in the simulation are represented by horizontal green lines. The predicted bound 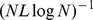 on mutation rates required for monomorphism is shown as a vertical dashed line. In (D)–(F) the fitted values of 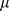, 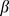, and 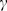 are shown as functions of the number of generations 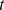 for the non-steady state simulations used in Fig. 2D–F. The sampling procedure, the maximum likelihood fit, and the representation of parameter predictions are the same as in (A)–(C).

We also infer parameters 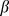, 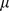, and 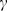 with a maximum likelihood fit. As expected, all parameters converge to the exact values in the monomorphic limit (Fig. 3A–C). When the population is not truly monomorphic, 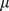 and 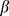 tend to be underestimated on average, with larger variation in inferred values (larger error bars in Fig. 3A,B). For 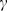, polymorphism has no clear bias on the average inferred value, although it also appears to increase the variation.

*Deviations from evolutionary steady state*. We perform another set of simulations to test the accuracy of our predictions in a population that has not yet reached steady state. We use the same fitness landscape and population size, but fix 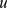 to 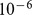, within the monomorphic limit. At each point in time (measured as the number of generations), we construct 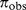 as described in Methods (Fig. 2D), and infer the fitness function (Fig. 2E). We also compute the TVD between the observed distribution 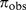 and the steady-state prediction (Fig. 2F). Over time 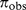 converges to the steady state (Eq. 3) and the TVD decays to zero, enabling accurate reconstruction of the fitness function in the region 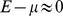 (although it still diverges from the exact function in the high-energy tail, where few sequences are available at steady state). The relaxation time is expected to be proportional to 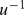, or 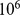 generations, which is in agreement with Fig. 2F. As the population reaches steady state, accurate inference of the fitness function parameters becomes possible (Fig. 3D–F). We see that parameters inferred from a population out of steady state tend to underestimate 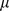 and 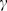, and overestimate 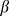.

### 2 Transcription factor binding sites in yeast

We now turn to considering the evolution of TF binding sites in *S. cerevisiae*. How well does *S. cerevisiae* satisfy the assumptions of our evolutionary model? First of all, *S. cerevisiae* is not a purely haploid organism but rather goes through haploid and diploid stages. In *S. paradoxus*, most of the reproduction is haploid and asexual with 1000 generations spent in the haploid stage for each generation in the diploid stage, and heterozygosity is low [45]. Based on the analysis of yeast genomes, wild yeast populations show limited outcrossing and recombination and are geographically distinct [46]. Thus, *S. cerevisiae* may be regarded as haploid to a reasonable approximation, with sufficient recombination during the diploid stages to unlink TF binding sites. This is consistent with our model, which assumes a haploid population and independent evolution of binding sites.

We next consider whether natural populations of *S. cerevisiae* are within the mutation rate limits required for monomorphism. The mutation rate for *S. cerevisiae* has been estimated to be 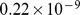 mutations per bp per cell division [45]. Assuming binding site loci of length 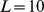, the bound on the effective population size 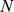 is 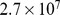, below which the population will be monomorphic. This is close to the estimated effective population size of *S. cerevisiae* of 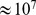 individuals [45], based on the analysis of neutral regions in the yeast genome. Thus it is plausible that *S. cerevisiae* population sizes are below or near the limit for monomorphism, justifying the use of Eq. 3. Furthermore, in *S. cerevisiae* and *S. paradoxus* the proportion of polymorphic sites in a population has been found to be about 0.001 [45], [47], [48], generally with no more than two alleles segregating at any one site [45]. According to this estimate, we expect about 1% of binding sites of length 10 bp to be polymorphic, corresponding to an average polymorphism of 1.01 in Fig. 2C.

For *S. cerevisiae*, the equilibration time estimate is 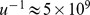 generations, or about 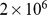 years with 8 generations per day [49]. This is several times less than the 5–10 million years of divergence time for the most recent speciation event with *S. paradoxus* [50]. Thus steady state may plausibly be reached over evolutionary times scales for a fast-reproducing organism like *S. cerevisiae*.

#### 2.1 Site-specific selection

We obtain curated binding site locations in *S. cerevisiae* from Ref. [31] and energy matrices (EMs) from Ref. [35], as described in Methods. Besides the assumptions of monomorphism and steady state, we also require an ensemble of binding sites evolving under universal selection constraints if we are to infer the fitness landscape using Eq. 5. A collection of sites binding to the same TF is an obvious candidate, since these sites all experience the same physical interactions with the TF. However, it is possible that selection is largely site-specific: rather than evolving on the same fitness landscape, different sites for the same TF may be under different selection pressures depending on which genes they regulate, their position on the chromosome, etc. For example, genes under strong selection might require very reliable regulation, so that their upstream binding sites are selected for tight binding to TFs. In less essential genes, the requirement of high-affinity binding might be relaxed. Before directly applying the evolutionary model, we investigate several of these site-specific scenarios to determine if any are supported by the available data. We perform several direct tests of site-specific selection by searching for correlations between site TF-binding energies and other properties of the site or the gene it regulates.

We classify fitness effects of genes using knockout lethality, which is available in the Yeast Deletion Database [39], [51]. This database classifies genes as either essential or nonessential based on the effects of gene knockout, and provides growth rates for nonessential gene knockouts under a variety of experimental conditions. We divide binding sites of each TF in our data set into two groups: those regulating essential genes and those regulating nonessential genes.

**Figure 4:**
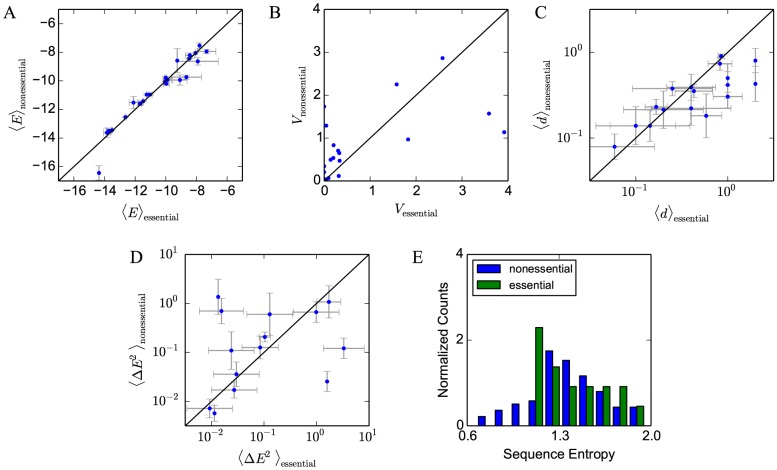
Tests of site-specific selection. We divide binding sites for each TF into two groups: those regulating essential genes and those regulating nonessential genes. (A) Comparison of mean binding energies of sites regulating essential (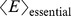) and nonessential genes (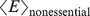) for each TF in the data set. (B) Comparison of variance in binding energies for sites regulating essential (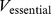) and nonessential (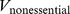) genes. (C) Mean Hamming distance between corresponding sites in *S. cerevisiae* and *S. paradoxus* for sites regulating essential (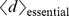) versus nonessential genes (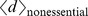). (D) Mean squared difference in binding energy between corresponding sites in *S. cerevisiae* and *S. paradoxus* for sites regulating essential (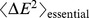) versus nonessential genes (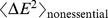). In (A)–(D), 25 TFs were used; black diagonal lines have slope one. In (A),(C),(D), vertical and horizontal error bars show the standard error of the mean in each group. Points lacking error bars have only one sequence in that group. (E) Normalized histogram of TF binding site sequence entropies, divided into 16 essential and 109 nonessential TFs, for 125 TFs in Ref. [31].

In Fig. 4A we compare mean binding energies of sites regulating essential genes with those regulating nonessential genes for each TF. Using a null model as described in Methods, we find no significant difference (at 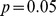 level) between the two groups of sites for any TF except RPN4 (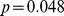). The mean 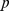-value of the null model over all TFs is 0.530; a small number of individually-significant 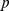-values is expected as a consequence of multiple testing. In Fig. 4B we compare the variance of the energy of the sites regulating essential and nonessential genes; sites regulating essential genes may be selected for more specific values of binding energy if precise regulation is required. We find no overall trend: for some TFs sites regulating essential genes have more energy variation than those regulating nonessential genes, but for other TFs the situation is reversed.

For the sites regulating nonessential genes, we also correlate the site binding energy with the growth rate of a strain in which the regulated gene was knocked out (Table S1, column B). The Spearman rank correlation between each site's binding energy and the regulated gene's effect on growth rate produces a mean 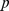-value of 0.562. We find no significant correlation for any TF at 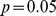 level except MSN2, with 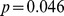.

It is also possible that regulation of highly-expressed genes may be more tightly controlled. Indeed, gene expression level is weakly, though significantly, correlated with gene essentiality [52]. We compare the binding energy of sites to the overall expression level of their regulated genes measured in mid-log phase yeast cells cultured in YPD [52] (Table S1, column C), and again find no correlation using the Spearman rank correlation except for DAL80 (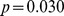), with mean 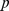-value of 0.537.

Another measure of the selection pressures on genes is their rate of evolution as measured by 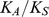, the ratio of nonsynonymous to synonymous mutations in a given gene between species. According to the neutral theory of evolution, genes which evolve slowly must be under higher selective pressure, and therefore the sites regulating them might likewise experience stronger selective pressures. As described in Methods, we measure the 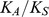 ratio between *S. cerevisiae* and *S. paradoxus* protein coding sequences, and compare it to the binding energy of the sites regulating those genes (Table S1, column D). We find very weak Spearman rank correlations for RPN4, GAT1, CAD1, and ATF2, all roughly with 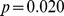. We find no other significant correlation at the 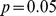 level, with a mean 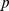-value of 0.404.

Similarly, one might expect sites regulating essential genes to be more conserved. However, we find that the average Hamming distance between corresponding binding sites in *S. cerevisiae* and *S. paradoxus* [31] is no different for sites regulating essential genes than for those regulating nonessential genes, as shown in Fig. 4C. Using the null model described in Methods, most TFs are above 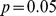 with the exceptions of PDR3 (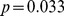), with an average 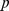-value of 0.651. Similarly, there is no significant difference in the binding energies of these orthologous sites as determined from the EMs, as shown in Fig. 4D, except for PDR3 (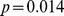), with mean 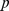-value of 0.691.

We can also consider how the essentiality of the TFs themselves affects the sequences of their binding sites; for example, essential TFs may constrain their binding sites to a more conserved sequence motif. We divide 125 TFs from Ref. [31] which had 10 or more sequences and for which essentiality information was available into 16 essential and 109 nonessential TFs using the Yeast Deletion Database [39], [51], and calculate the sequence entropy of binding sites for each TF. The distribution of sequence entropies in Fig. 4E shows no significant difference between essential and nonessential TFs (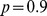 for the null model).

Finally, it is possible that sites experience different selection pressures depending on their distance to the transcription start site (TSS). Again, we find no significant correlations between binding energy and distance to the TSS: Spearman rank correlation yields mean 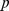-value of 0.560 and all 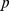-values above 0.05 except RPN4 (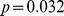) (Table S1, column E). Overall, we find no systematic evidence that site-specific properties of binding sites determine their binding energies. These findings are in broad agreement with a previous report [22], which suggested that site-specific selection can be ruled out because of the significant variation in binding affinity between orthologous sites of different species, which was found to be consistent with the variance predicted by a model including only drift and site-independent selection.

#### 2.2 Inference of biophysical fitness landscapes

The above analysis indicates that the evolution of binding site energies does not depend significantly on site-specific effects, suggesting that more universal principles govern the observed distribution of sites binding a given TF. Thus, we will fit a single fitness function to a collection of TF-bound sites via Eqs. 3 and 8. Of the 25 TFs considered in the previous section, here we focus on 12 TFs with 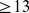 unique binding site sequences.

**Figure 5:**
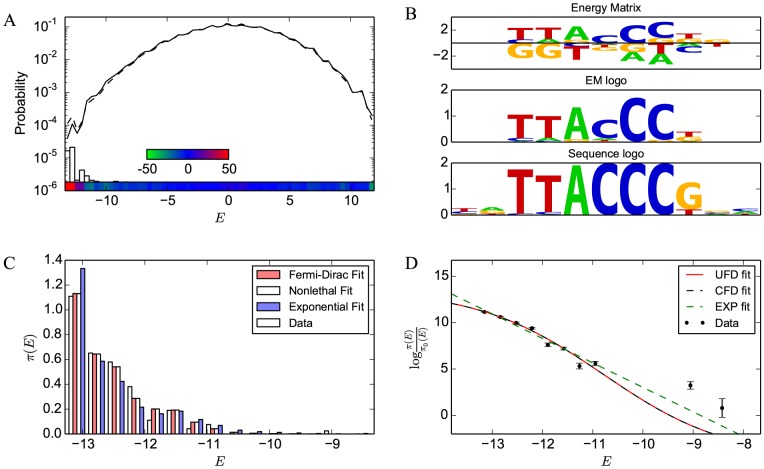
Parametric inference of REB1 fitness landscape. (A) Histogram of energies of intergenic sites calculated using the REB1 energy matrix (dashed line) and the neutral distribution of sequence energies expected from the mono- and dinucleotide background model (solid line; see Methods for details). The color bar on the bottom indicates the percent deviation between the two distributions (red is excess, green is depletion relative to the background model). The vertical bars show the distribution of functional sites [31], which correspond to the low-energy excess in the distribution of intergenic sites. (B) From top to bottom: REB1 energy matrix, the sequence logo obtained from the energy matrix by assuming a Boltzmann distribution at room temperature at each position in the binding site (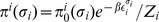), and the sequence logo based on the alignment of observed REB1 genomic sites. (C) Histogram of binding site energies and its prediction based on the three fits (Eq. 5). (D) Fitness function inference. Dots represent data points (as in Fig. 6); also shown are the unconstrained fit to the Fermi-Dirac function of Eq. 7 (“UFD”; solid red line), constrained fit to Eq. 7 with 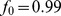 (“CFD”; dashed black line), and fit to an exponential fitness function (“EXP”; dashed green line). Error bars in (D) are calculated as in Fig. 6.

First we derive the neutral distribution 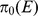 of site energies based on mono- and dinucleotide frequencies obtained from intergenic regions of the *S. cerevisiae* genome, as described in Methods. It has been suggested that 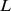-mers not functioning as regulatory sites (e.g., located outside promoters) may be under evolutionary pressure not to bind TFs [53]; however, consistent with previous reports [22], [54], we find that sequences sampled from the intergenic regions of the genome are close to the neutral distribution expected from mono- and dinucleotide frequencies, except for the expected enrichment at low energies due to functional binding sites. This distribution is shown in Fig. 5A for REB1 and in Table S2, column B for all other TFs.

**Figure 6:**
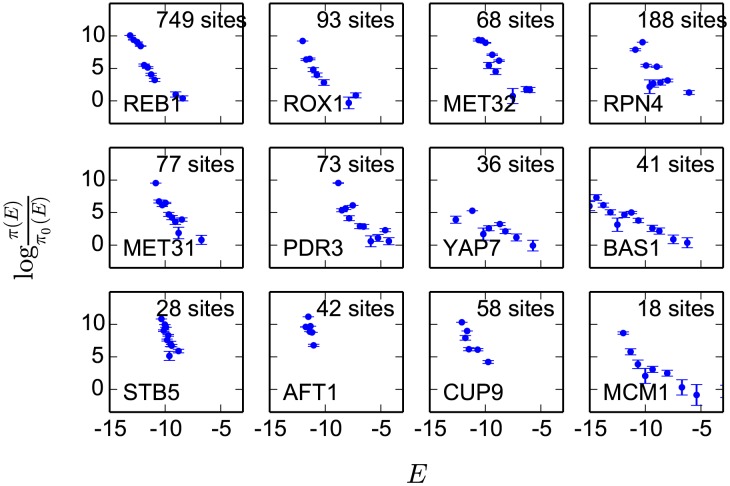
Qualitative behavior of fitness landscapes. Shown are plots of 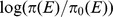 for 12 TFs, which, according to Eq. 8, equals the logarithm of fitness up to an overall scale and shift. For each TF, sequences are grouped into 15 equal-size energy bins between the minimum and maximum energies allowed by the energy matrix. Shown also are the total number of binding sites for each TF. Error bars are calculated as 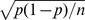, where 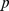 is the fraction of sites falling into a given bin out of 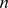 total sites, as would be expected if the sequences were randomly distributed according to the observed distribution.

Assuming the observed set of binding site energies for a TF adequately samples the distribution 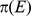, we can use our estimate of the neutral distribution 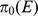 in Eq. 8 to reconstruct the fitness landscapes as a function of TF binding energy up to an overall scale and shift (Fig. 6). Although the fitness functions may be noisy due to imperfect sampling of 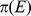, they nevertheless provide important qualitative insights. In particular, in all cases fitness decreases monotonically as binding energy increases, indicating that stronger-binding sites are more fit. That is, we observe no fitness penalty for binding too strongly, at least within the range of energies spanned by 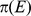.

*Fitted Fermi-Dirac landscapes*. For each TF we perform a maximum-likelihood fit of the binding site data to the distribution in Eq. 3 with the Fermi-Dirac landscape of Eq. 7 (Fig. 5 for the REB1 example, Table S2 for all other TFs; see Methods for details). The model of Eq. 7 has four fitting parameters: 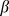, 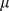, 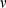, and 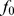. However, as shown in Sec. 1.3, in the 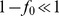 limit the fitness function depends on 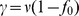 rather than 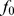 and 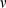 separately. Thus we also carry out constrained “non-lethal” Fermi-Dirac fits in which 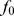 is fixed at 0.99. Note that due to the 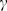-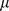 degeneracy, in some cases 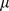 effectively fits to the limiting cases 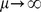 or 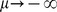 rather than a specific value. Because we only fit in the range 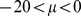 (see Methods), a value of 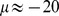 shows that the fit is subject to the 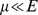 degeneracy, while 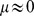 shows that it is subject to the 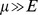 degeneracy. As mentioned above, the input to each fit is a collection of genomic TF binding sites 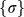 [31] and the EM predicted on the basis of high-throughput *in vitro* TF-DNA binding assays [35]. The EM allows us to assign a binding energy 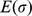 to each site.

A summary of maximum-likelihood parameter values for all TFs is shown in Tables 1 and S2, column D. The variation of log-likelihood with fitting parameters is shown in Table S2, columns G and H. Since for many TFs relatively few binding sites are available, in Table S3 we evaluate the goodness of fit using randomly chosen subsets of binding sites and Hessian analysis. Six of the TFs (REB1, ROX1, MET32, PDR3, CUP9, and MCM1) are in the 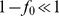 regime where only 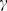 can be inferred unambiguously. Indeed, non-lethal Fermi-Dirac fits with 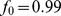 yield very similar values of log-likelihood and 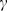 (Table S2, column D). In all of these cases, 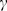 is considerably greater than 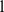, implying that selection is strong compared to drift and the effective population size is large (the 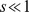, 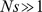 regime in population genetics).

**Table 1:** Summary of unconstrained Fermi-Dirac landscape fits to TF binding site data.

TF	*f*_0_	*γ* = *ν*(1−*f*_0_)	*β* (in (kcal/mol)^−1^)	*E*−*μ*
REB1	0.999	18.2	0.794	≈0
ROX1	0.993	335	0.398	>0
MET32	0.973	133	0.251	>0
RPN4	1.01×10^−4^	1.58	2.0	≈0
MET31	4.54×10^−5^	1.58	1.58	≈0
PDR3	0.988	242	0.251	>0
YAP7	4.54×10^−5^	1.58	1.0	≈0
BAS1	4.54×10^−5^	2.51	0.501	≈0
STB5	0.401	150	0.316	<0
AFT1	4.54×10^−5^	3.98	5.01	≈0
CUP9	0.978	219	0.316	>0
MCM1	0.998	83.1	0.251	>0

Columns show maximum-likelihood value of 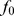, 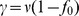, and 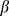. The last column shows whether most binding site energies 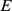 are lower than the inferred chemical potential 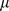, near it, or above it (see Table S2 for details).

Five TFs (RPN4, MET31, YAP7, BAS1, and AFT1) have very small values of 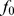 (Table 1), indicating that on average, removing their binding sites is strongly deleterious to the cell. In these cases, the global maximum occurs in the vicinity of 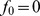, away from the degenerate region of parameter space (Table S2, column H, insets). Note however that the likelihood surface is always degenerate in the region of parameter space with 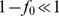 and 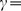 constant; this is true even when the global maximum likelihood does not occur in that region, as observed for these five TFs. Since 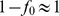, 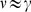, which is a small value in all five cases (Table 1). Given the strength of selection, small effective population sizes (which indicate that genetic drift is strong) are necessary to reproduce the observed variation in binding site sequences. Finally, sites for STB5 have an intermediate value of 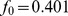, which means they are under strong selection but are not necessarily essential.

The fits to the Fermi-Dirac fitness landscapes also provide estimates of the effective inverse temperature 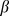 (Table 1). The inferred values of 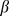 can be compared to the physical value at room temperature, 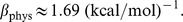 Ten of the TFs (REB1, ROX1, MET32, MET31, PDR3, YAP7, BAS1, STB5, CUP9, MCM1) have 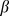
's lower than the physical value, while in the other two (RPN4, AFT1) 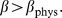 In most TFs the fitted inverse temperature 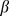 is far from its physical counterpart, although in several cases the likelihood function is fairly flat in the vicinity of the peak, indicating that a wider range of 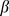 values is admissible (Table S2, column G).

The inferred value of 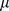 relative to the distribution of energies 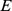 of the binding sites tells us in which qualitative regime of the Fermi-Dirac fitness landscape the sites lie. For five TFs (ROX1, MET32, PDR3, CUP9, MCM1) 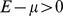, so the sites reside on the exponential tail of the landscape; since 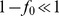 as well, they are subject to the 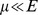 degeneracy. For a group of six TFs (REB1, RPN4, MET31, YAP7, BAS1, AFT1), 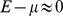, so that the sites lie on the bound-unbound threshold. In this regime, changing the energy of the site through mutations may lead to a large change in fitness. Finally, 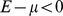 for STB5, so the sites lie on the high-fitness plateau and are subject to the 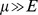 degeneracy. The degeneracies in 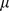 are also illustrated in Table S2, column G.

What does 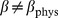 say about the nature and strength of selection? We address this question using the local selection coefficient, 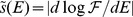 (Eq. 13). The magnitude of the selection coefficient depends qualitatively on both 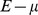 and whether 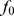 is zero or nonzero (Fig. 1). For five of the TFs (ROX1, MET32, PDR3, CUP9, MCM1), 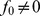, 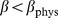, and 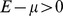. Thus these TFs are in a regime where decreasing 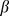 strengthens selection (Fig. 1F). In other words, selection is stronger for these binding sites than expected from purely biophysical considerations. For RPN4 and AFT1, 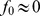, 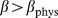, and 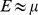. Hence 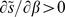, and selection is again stronger than expected. STB5 exhibits 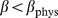 and lies on the high fitness plateau (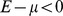), and thus selection is also stronger than expected. In contrast, REB1, MET31, YAP7, and BAS1 exhibit 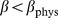 and lie on the threshold 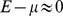, and hence selection is weaker than expected in these four cases.

*Fitness landscape model selection*. Since the constrained Fermi-Dirac fits have one fewer adjustable parameter than the unconstrained fits, it is more consistent to do model selection on the basis of the Akaike information criterion (adjusted for finite-size samples) [55] rather than log-likelihoods:
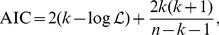
(15)where 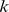 is the number of fitting parameters, 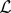 is the likelihood, and 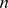 is the number of data points. For each model we can calculate the AIC, which accounts for both the benefits of higher log-likelihood and the costs of additional parameters. The model that provides the best fit for the fewest parameters will have the lowest AIC value.

Table 2 shows the AIC differences between the unconstrained Fermi-Dirac fits (UFD, 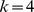) and the constrained Fermi-Dirac fits with 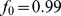 (CFD, 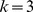) for each TF. Positive AIC differences indicate that UFD is more favorable. We also calculate the Akaike weights 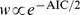, which give the relative likelihood that a given model is the best [55].

**Table 2:** Comparison of fitness function models.

TF	AIC_CFD_–AIC_UFD_	AIC_EXP_–AIC_UFD_	*w*_UFD_	*w*_CFD_	*w*_EXP_
REB1	3.054	35.734	0.822	0.178	1.43×10^−8^
ROX1	−2.042	34.753	0.265	0.735	7.53×10^−9^
MET32	−2.233	10.540	0.246	0.752	0.001
RPN4	5.674	19.959	0.945	0.055	4.38×10^−5^
MET31	−1.469	−3.869	0.100	0.208	0.692
PDR3	−2.124	6.123	0.254	0.734	0.012
YAP7	−2.049	10.722	0.264	0.735	0.001
BAS1	−2.107	1.061	0.224	0.644	0.132
STB5	−2.732	−7.145	0.025	0.097	0.879
AFT1	−2.069	6.096	0.259	0.729	0.012
CUP9	−2.251	1.560	0.220	0.679	0.101
MCM1	−3.343	−0.175	0.135	0.718	0.147

For each TF, we show the AIC differences between the unconstrained Fermi-Dirac fit (“UFD”), the constrained Fermi-Dirac fit with 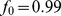 (“CFD”), and the exponential fit (“EXP”). Also shown are Akaike weights 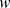, which indicate the relative likelihood of each model.

For five of the six TFs in the 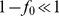 regime, the constrained Fermi-Dirac fits perform somewhat but not drastically better than the unconstrained Fermi-Dirac fits (Table 2). Indeed, the Akaike weights for the constrained Fermi-Dirac fits exceed the full fits for these TFs consistently by about a factor of 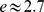, since their raw likelihoods are essentially equivalent and they only differ in the number of fitted parameters 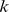. Out of the five TFs for which 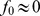, YAP7, BAS1, and AFT1 fit slightly better to the constrained Fermi-Dirac, suggesting that their small fitted values of 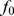 are not significant. For RPN4 the AIC analysis shows preference for the fits with low 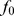; however RPN4 is listed as nonessential in the Yeast Deletion Database [39], [51], suggesting either an inconsistency in our analysis or that growth media tested in Refs. [39], [51] do not reveal essentiality of this TF.

We may also consider a purely exponential fitness landscape of the form 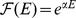. The reasons for including this case are threefold. First, exponential fitness emerges in the limit 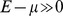 of the Fermi-Dirac landscape, the regime into which many of the TF binding sites fall. Second, the fitness landscapes in Fig. 6 appear close to linear on the logarithmic scale, implying that to a good approximation fitness depends exponentially on energy. Third, the model has just one fitting parameter, making it a useful null case for AIC evaluation.

The steady-state distribution 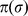 with exponential fitness is given by
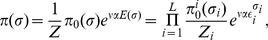
(16)where 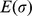 is given by Eq. 2, 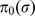 is the neutral probability of sequence 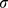, 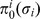 is the background probability of nucleotide 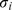 at position 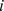, and 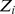 is a single-site partition function: 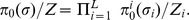 Here we assumed that the background probability of a sequence is a product of probabilities of its constituent nucleotides. In this case, positions in the binding site decouple and the distribution of sites 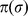 completely factorizes. The assumption of factorization underlies the common practice of inferring energy matrices from log-odds scores of observed genomic binding sites [32]. The log-odds score of a nucleotide 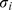 is defined as
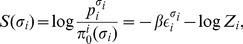
(17)where 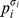 is the probability of observing base 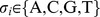 at position 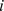 within the set of known sites, 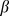 is an effective inverse temperature, and 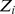 is the normalization constant. Equation 17 shows that the log-odds score, which is computed using observed nucleotide probabilities, is equivalent to 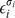 (up to an overall scale and shift) under the assumption of site independence.

We can quantitatively compare the exponential fitness landscape with the unconstrained and constrained Fermi-Dirac landscapes using the Akaike information criterion, Eq. 15. The AIC analysis shows that the exponential landscape is significantly poorer than the Fermi-Dirac landscape in all cases except MET31 (Table 2), where the exponential fit is marginally better than the Fermi-Dirac fits, and STB5, where the exponential landscape performs much better than the Fermi-Dirac models. This observation provides statistical support for the fitness landscapes of Fermi-Dirac type and for the non-lethality of deleting most TFs (the exponential fitness decays to zero rather than a nonzero 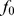 found in most of our Fermi-Dirac fits).

## Discussion

In this work, we have considered how fitness of a single-cell eukaryote *S. cerevisiae* is affected by interactions between TFs and their cognate genomic sites. Changing the energy of a site or creating new sites in gene promoters may change how genes are activated and repressed, which in turn alters the cell's chances of survival. Under the assumptions of a haploid monomorphic population in which the evolution of binding sites has reached steady state, the fitness landscape as a function of TF binding energy can be inferred from the distribution of TF binding sites observed in the genome, using a biophysical model which assigns binding energies to sites. We use a simple energy matrix model of TF-DNA energetics in which the energy contribution of each position in the site is independent of all the other positions. The energy matrix parameters are inferred from a high-throughput data set in which TF-DNA interactions were studied *in vitro* using a microfluidics device [35]. We consider two types of fitness functions: Fermi-Dirac, which appears naturally from considering TF binding as a two-state process (Eq. 1), and exponential, which is motivated by the observation that for many TFs, the logarithm of fitness appears to decrease linearly as energy increases.

A single fitness landscape for all genomic binding sites of a given TF can only exist in the absence of site-specific selection. Indeed, it is possible that TF sites experience different selection pressures depending on the genes they regulate: for example, sites in promoters of essential genes may be penalized more for deviating from the consensus sequence. In this case, the fitness function is an average over all sites which evolve under different selection constraints: as an extreme example, consider the case where each site 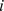 has a Fermi-Dirac fitness function (Eq. 7) with different parameters 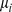, 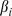, and 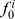. The resulting observed distribution of energies would then be the average of the distributions predicted by Eq. 5:
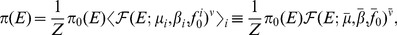
(18)which defines the “average” fitness function with effective parameters 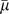, 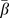, 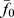, 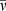. Thus the fit can be carried out even in the presence of site-dependent selection, but the fitted parameters correspond to fitness functions of individual sites only in an average sense.

To gauge the importance of site-specific selection in TF binding site evolution, we have performed several statistical tests aimed at discovering correlations between binding site energies and biological properties of the sites and the genes they regulate. These tests considered gene essentiality, growth rates of strains with nonessential genes knocked out, gene expression levels, 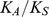 ratios based on alignments with *S. paradoxus*, and the distance of the site to the TSS. We find no consistent correlations among these properties, indicating that for a given TF, the evolution of regulatory sites is largely independent of the properties of regulated genes.

Previously, low correlations have been observed between essentiality and conservation of protein and coding sequences [56]–[62], which has fueled considerable speculation as it contradicts the prediction of the neutral theory of evolution that higher selection pressures lead to lower evolutionary rates. It has also been found that the growth rates of strains with nonessential genes knocked out are significantly (though weakly) correlated with conservation of those genes [63]. It has therefore been suggested that selection pressures are so strong that only the most nonessential genes experience significant genetic drift [56]. Previous studies have also found that gene expression levels are a more reliable (though still very weak) predictor of selection pressures than essentiality [60], but we do not find this to be the case for TF binding sites, nor do we observe a consistently significant correlation between gene expression levels and TF binding energies.

Available data does not rule out the possibility of time-dependent selection in combination with forms of site-dependent selection for which we have not accounted. In this scenario, the variation in site binding affinity is not due to genetic drift, but to variable selection pressures across sites and over time, such that the sites are strongly tuned to particular binding energies which change from locus to locus. Indeed, there is evidence that there is frequent gain and loss of TF binding sites and that the gene regulatory network is highly dynamic [64]–[70]. However, it is possible that rapid turnover of binding sites in eukaryotes may be due to evolution acting on whole promoters rather than individual binding sites. Many promoters contain multiple binding sites for a single TF, and it may be that while individual binding sites are lost and gained frequently, the overall binding affinity of a promoter to a TF may be held constant [71]–[73]. Our evolutionary model can account for this scenario using a promoter-level fitness function, which we intend to consider in future work.

Out of 12 TFs with sufficient binding site data, five have 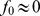, indicating a large fitness penalty for deleting such sites. However, this conclusion is strongly supported by the AIC differences between unconstrained and non-lethal Fermi-Dirac fits for only one TF, RPN4 (Table 2). RPN4 is classified as nonessential in the Yeast Deletion Database. It may be that this misclassification is due to a mismatch between genomic sites, in which the core 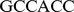 motif is preceded by 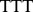, and the energy matrix in which the binding energies upstream of the core motif are non-specific (Table S2). We also classify REB1 and MCM1 binding sites as nonessential, although knocking out these TFs is lethal in yeast. This discrepancy may be due to a minority of essential sites being averaged with the majority of nonessential sites to produce a single fitness function, as described above. In addition, although a penalty for deleting any single site may be small, the cumulative penalty for deleting all sites (or, equivalently, deleting the TF) may be lethal.

We find that in 10 out of 12 cases, fitting an exponential fitness function is less supported by the data than fitting a Fermi-Dirac function (Table 2). This is interesting since constructing a position-specific weight matrix by aligning genomic sites is a common practice which implicitly assumes factorization of exponential fitness and independence of each position in the binding site. Our results show the limitations of this approximation. It is important to note that a key difference between the Fermi-Dirac fitness landscape and the exponential landscape is that the former contains magnitude epistasis [8], [25] (i.e., the magnitude of a mutation's fitness effect depends on the rest of the site sequence), while the latter is non-epistatic. Thus, our results indicate that epistasis is widespread in the evolution of TF binding sites [22].

Finally, we find that depending on the TF, the distribution of TF binding energies may fall on the exponential tail, across the threshold region, or on the saturated plateau where the sites are always occupied (Table 1). In the first two categories, variation of TF concentration in the cell will lead to graded responses, which may be necessary to achieve precise and coordinated gene regulation. In the third regime, TF binding is robust and not dynamic. We also find that the fitted inverse temperature 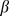 is typically not close to the value based on room temperature (Table 1). In particular, our analysis of the variation of selection strength with 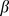 indicates that selection appears to be stronger for most TFs than expected from pure biophysical considerations, suggesting the presence of additional selection pressures beyond those dictated by the energetics of TF-DNA binding.

## Methods

### Distribution of monomorphic population genotypes in evolutionary steady state

In the limit 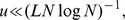 where 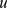 is the mutation rate per nucleotide, 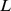 is the number of nucleotides in a locus, and 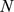 is an effective population size, mutations are sufficiently rare that each new mutation either fixes or goes extinct before the next one arrives [41]. Thus populations evolve by sequential substitutions of new mutations at a locus, which consist of a single new mutant arising and then fixing. The rate at which a given substitution occurs is thus given by the rate of producing a single mutant times the probability that the mutation fixes [36]:
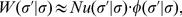
(19)where 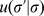 is the mutation rate from genotype 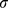 to 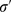 and 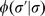 is the probability that a single 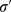 mutant fixes in a population of wild-type 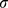. We will assume that 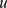 is nonzero only for sequences 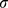 and 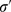 differing by a single nucleotide.

Given an ensemble of populations evolving with these rates, we can define 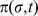 to be the probability that a population has genotype 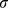 at time 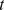. This probability evolves over time according to the master equation
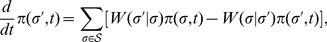
(20)where 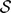 is the set of all possible genotypes at the locus of interest. This Markov process is finite and irreducible, since there is a nonzero probability of reaching any sequence from any other sequence in finite time. Hence it has a unique steady-state distribution 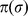 satisfying [74]

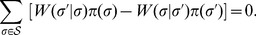
(21)For population models obeying time reversibility, we can show that the steady-state distribution 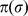 must have the form in Eq. 3 [38]. We assume the fixation probability 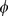 depends only on the ratio of mutant to wild-type fitnesses: 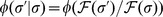. This occurs in most standard population models and is expected whenever only relative fitness matters (e.g., when the total population size is constant). If the population dynamics are time reversible, the substitution rates and steady state must obey the detailed balance relation 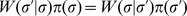. Assuming the neutral dynamics also obey detailed balance, 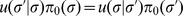, we can show that
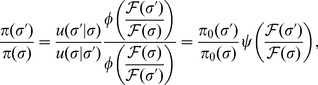
(22)where 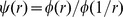. Equation 22 implies that 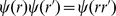, leading to 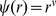 for some exponent 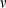. It can be shown that 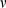 must be proportional to the effective population size [38]; for the Wright-Fisher model, 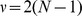. Now Eq. 3 follows from
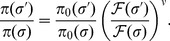
(23)This form of the steady state assumes only time reversibility and dependence on fitness ratios; otherwise, any form of the fixation probability must satisfy it. While many population models do not obey time reversibility exactly, it can be shown that even these irreversible models satisfy Eq. 3 to a very good approximation [38].

### Maximum-likelihood fits of fitness function parameters

For a given TF, let 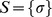 be the set of binding site sequences and 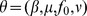 the parameters of the fitness function (Eq. 7). The log-likelihood is given by
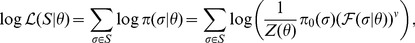
(24)where 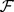 is the fitness function, and 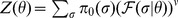 is the normalization.

Because the log-likelihood function has degenerate or nearly-degenerate regions in the parameter space of 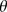, instead of maximizing by gradient ascent we obtain a global map of the likelihood by calculating the function over a mesh of points in the following parameter domain: 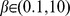 generated from 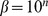 for 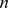 running from 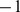 to 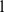 in steps of 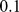; 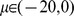 in steps of 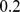; 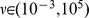 generated from 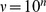 for 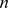 running from 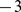 to 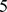 in steps of 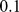; and 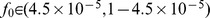 generated from 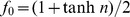 for 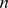 running from 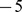 to 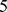 in steps of 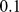. Our predicted maximum is the maximum likelihood point in the mesh, which is sufficiently fine to estimate all fitting parameters. We have made the code for this procedure and for the analysis of site-specific selection available at www.physics.rutgers.edu/~morozov/publications.html.

### Simulations

We consider a haploid asexual Wright-Fisher process [43]. The population consists of 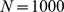 organisms, each with a single locus of 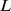 nucleotides. The new generation is created by means of a selection step and a mutation step. In the selection step, sequences from the current population are sampled with replacement, weighted by their fitness, to construct a new population of size 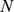. In the mutation step, each position in all sequences is mutated with probability 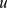. For simplicity, the mutation rates between all pairs of nucleotides are the same.

We characterize the difference between the distribution expected by our model, 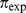 (Eq. 3), and the distribution observed in simulations, 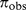, using the total variation distance (TVD):
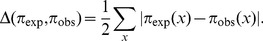
(25)The TVD ranges from zero for identical distributions to unity for completely non-overlapping distributions. We calculate the TVD for the distributions in energy space, where the sum in Eq. 25 is over discrete energy bins (we bin the observed sequences by energy by dividing the range from the minimum to the maximum sequence energy for a particular energy matrix into 100 bins of equal size).

We begin by randomly generating the energy matrix parameters 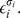 Each 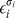 in the energy matrix is sampled from a uniform distribution and then rescaled such that the distribution of all sequence energies has standard deviation of 1.0. This is achieved by dividing all entries in the energy matrix by a factor 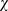:
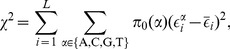
(26)where 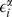 is the energy matrix element for base 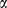 at position 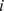, 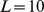 is the binding site length, 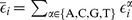 is the average energy contribution at position 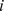, and 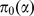 is the background probability of nucleotide 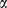 (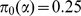 for all 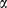 in our simulations). It can be shown that 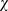 is the standard deviation of the random sequence energy distributution, which is approximately Gaussian [11]. We generate the energy matrix once and use it in all subsequent simulations and maximum likelihood fits.

We perform the Wright-Fisher simulations in a range of mutation rates from 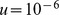 to 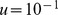 with a “non-lethal” Fermi-Dirac fitness function (Eq. 7 with 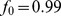, 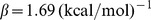, and 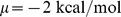). We run 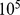 simulations for each mutation rate for 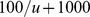 steps, enough to reach steady state. Each simulation starts from a monomorphic population with a randomly chosen sequence. We construct the steady state distribution for each mutation rate by randomly choosing a single sequence from the final population of each simulation. Collected across all simulations, these are used to construct a distribution of sequences at each mutation rate. Additionally, we record the average final number of unique sequences at each mutation rate.

We perform another set of Wright-Fisher simulations with the same fitness function and energy matrix as above, and 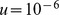. We run 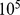 simulations, each starting from the same monomorphic population with a specific sequence of 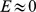. At regular intervals in each simulation, we record a randomly chosen sequence from the population. Collected across all simulations, these are used to construct a distribution of sequences at each point in time.

### Binding site and energy matrix data

We obtain curated binding site locations for 125 TFs from Ref. [31], which provides a posterior probability that each site is functional based on cross-species analysis. We only consider sites with a posterior probability above 0.9. For this analysis, we use the Saccharomyces Genome Database R53-1-1 (April 2006) build of the *S. cerevisiae* genome.

We obtain position-specific affinity matrices (PSAMs) for a set of 26 TFs from an *in vitro* microfluidics analysis of TF-DNA interactions [35]. This study provides PSAMs for each TF determined using the MatrixREDUCE package [34]. We convert the elements of the PSAM 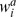 to energy matrix elements using 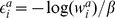, where 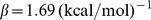 at room temperature. For each of these 26 TFs, genomic sites are available in Ref. [31], although we neglect PHO4 since it does not have any binding sites above the 0.9 threshold of Ref. [31], leaving us with 25 TFs for which both an energy matrix and a set of genomic binding sites are available. We align the binding site sequences from Ref. [31] to the corresponding energy matrices, choosing the alignment that produces the lowest average binding energy for the sites.

### Essentiality data

The Yeast Deletion Database classifies genes as essential, tested (nonessential), and unavailable, which number 1156, 6343, and 529, respectively [39], [51]. For each essential or tested gene, we determine all TF binding sites less than 700 bp upstream of the gene's transcription start site (on either strand), which we designate as the sites regulating that gene. Growth rates for nonessential knockout strains are provided under YPD, YPDGE, YPG, YPE, and YPL conditions, relative to wild-type. We choose the lowest of these growth rates to represent the fitness effect of the knockout.

To measure the rate of nonsynonymous substitutions, we align the non-mitochondrial, non-retrotransposon ORFs taken from the Saccharomyces Genome Database R64-1-1 (February 2011) build [75] of *S. cerevisiae* to those of *S. paradoxus* using ClustalW [76]. We measure the rate of nonsynonymous mutations using PAML [77]. We ran PAML with a runMode of −2 (pairwise comparisons) and the CodonFreq parameter (background codon frequency) set to 2; we also tested CodonFreq set to zero and obtained very similar results. We find the rate of nonsynonymous substitutions to be 0.04, and a Spearman rank correlation of 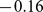 (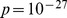) between growth rate of knockouts and the nonsynonymous substitution rate of the knocked-out gene. This is consistent with the results of Ref. [58], which found the rate of substitutions to be 0.04 and the rank correlation between growth rate and substitution rate to be 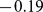 (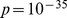).

To compare binding energy to evolutionary conservation, we calculate the mean Hamming distance between *S. cerevisiae* sites and corresponding sites in *S. paradoxus* [31]. To test for significance in the difference of mean energies and Hamming distances of sites regulating essential and nonessential genes, we use a null model which assumes that the sites were randomly categorized into essential and nonessential. We randomly choose a subset of the sites in our dataset to be “nonessential,” equal in size to the number of sites regulating nonessential genes as classified by the Yeast Deletion Database. By repeating this procedure 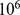 times, we build a probability distribution for the difference in the means of the nonessential and essential groups. The 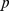-value is the probability of obtaining a difference in the means greater than or equal in magnitude to the empirically measured value.

### Neutral binding site energy distributions

We construct the neutral probability 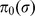 of a sequence 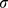 with length 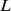 as
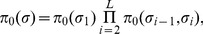
(27)where 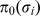 is the background probability of a nucleotide 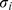, and 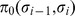 is the background probability of a dinucleotide 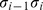. These probabilities are determined from mono- and dinucleotide frequencies in the intergenic regions of the *S. cerevisiae* genome (build R61-1-1, June 2008). We project 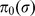 into energy space using Eq. 2 to obtain 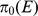, the neutral distribution of binding energies for sequences of length 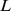.

If intergenic sequences evolve under no selection with respect to their TF-binding energy, the neutral distribution of site energies should closely match the actual distribution of 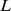-mer sequences obtained from intergenic regions. Table S2, column B shows that these two distributions match very well except at the low-energy tail, which is enriched in functional binding sites. Note that accounting for dinucleotide frequencies is important; mononucleotide frequencies alone are insufficient to reproduce the observed distribution [54].

## Supporting Information

Table S1
**Full summary of tests for site-specific selection.** For 25 TFs we compute TF-DNA interaction energies (in kcal/mol) for each site. Columns from left to right: (A) Essentiality of the TF according to the Yeast Deletion Database; total number of binding sites for each TF; total number of sites with unique sequences. The table lists how many essential and nonessential genes are regulated by each TF, and how many of these genes have gene expression and *S. paradoxus* 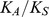 ratio data. We also report the mean energy 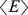 and the variance 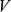 of sites regulating both essential and nonessential genes, and mean squared energy difference 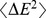 and mean Hamming distance 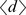 between *S. cerevisiae* and *S. paradoxus* sites regulating essential and nonessential genes. We show 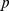-values for the significance of the difference between these two classes of sites (see Methods). (B) Growth rate in strains with nonessential gene knockouts versus energy of TF binding sites regulating the knockout genes. (C) Gene expression versus energy of TF sites regulating the genes. (D) Ratio of nonsynonymous to synonymous substitutions (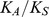) in genes versus energy of their TF regulatory sites. (E) Distance between each binding site and the closest transcription start site (TSS) versus the energy of the site. For (B)–(E) we report the Spearman rank correlation 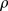 between each property and site energy, along with the 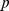-value of its significance (see Methods).(PDF)

Table S2
**Summary of fitness landscape fits to TF binding site data.** We consider 12 TFs which have at least 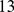 unique binding site sequences. Each row corresponds to a TF, ranked in the decreasing order of the number of unique binding site sequences. Columns, from left to right: (A) Summary of TF binding site data. (B) Same as Fig. 5A. (C) Same as Fig. 5B. (D) Fitted values of fitness landscape parameters, maximized log-likelihoods, and AICs for the unconstrained fit to the Fermi-Dirac function of Eq. 6 (“UFD”), constrained fit to the Eq. 6 with 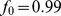 (“CFD”), and fit to an exponential fitness function (“EXP”). (E) Same as Fig. 5C. (F) Same as Fig. 5D. (G) Left panel: Log-likelihood of the unconstrained Fermi-Dirac model as a function of the effective chemical potential 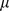. For reference, the distribution of functional binding site energies (same as in (B)) is shown on top. Right panel: Log-likelihood as a function of the effective inverse temperature 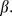 For reference, the inverse room temperature 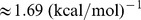 is shown as the vertical dashed line. To generate the log-likelihood plots, 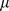 or 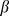 were scanned across a range of values while all the other parameters were re-optimized for each new value of 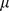 or 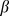. (H) Heatmap of log-likelihood as a function of 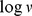 and 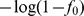 (note that 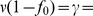 constant corresponds to a straight line with slope 1 in these coordinates). For likelihoods that have a maximum near 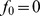, insets show a zoomed-in view. To generate the log-likelihood heatmaps, 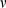 and 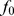 were scanned across the region shown while the other parameters (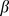 and 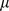) were re-optimized at each point separately.(PDF)

Table S3
**Estimates of fitting error.** For the 12 TFs in Table 1, we analyze the quality of fit. Columns, from left to right: (A) Eigenvalues and eigenvectors of the Hessian of the likelihood function around the fit maxima. Eigenvectors of the Hessian represent principal directions and the corresponding eigenvalues represent the curvature in those directions, which should be negative at a local maximum. Positive eigenvalues occur if the maximizer did not reach a maximum. Here, the degeneracy represented by 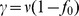 is apparent as many fits have an eigenvalue close to zero (flat) or even slightly positive in the direction 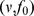. For fits subject to the 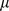-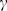 degeneracy, one can see a second low eigenvalue corresponding to the 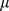 direction. For computational reasons the Hessian is evaluated using transformed variables 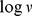, 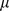, 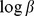, and 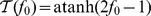. (B) For each TF, 64 subsets of the full data set were generated by randomly selecting half of the binding sites in the full data set. Maximum likelihood fits were carried out as for the full data set, except that to reduce computation time the grid spacing in the initial four dimensional parameter search was doubled. Shown here are histograms of the resulting parameters. Red dashed lines indicate the maximum likelihood value of each parameter obtained from the full data set.(PDF)

## References

[1] Wright S (1932) The roles of mutation, inbreeding, crossbreeding and selection in evolution. Proc 6th Int Cong Genet 1: 356–366.

[2] Orr HA (2009) Fitness and its role in evolutionary genetics. Nat Rev Genet 10: 531–539. 19546856 10.1038/nrg2603 PMC2753274

[3] Szendro IG, Schenk MF, Franke J, Krug J, de Visser JA (2013) Quantitative analyses of empirical fitness landscapes. J Stat Mech P01005.

[4] Kauffman SA, Weinberger ED (1989) The NK model of rugged fitness landscapes and its application to maturation of the immune response. J Theor Biol 141: 211–245. 2632988 10.1016/s0022-5193(89)80019-0

[5] Kauffman S (1993) The Origins of Order: Self-Organization and Selection in Evolution. (Oxford University Press, New York).

[6] Franke J, Klozer A, de Visser JA, Krug J (2011) Evolutionary accessibility of mutational pathways. PLoS Comput Biol 7: e1002134. 21876664 10.1371/journal.pcbi.1002134 PMC3158036

[7] Weinreich DM, Delaney NF, DePristo MA, Hartl DL (2006) Darwinian evolution can follow only very few mutational paths to fitter proteins. Science 312: 111–114. 16601193 10.1126/science.1123539

[8] Poelwijk FJ, Kiviet DJ, Weinreich DM, Tans SJ (2007) Empirical fitness landscapes reveal accessible evolutionary paths. Nature 445: 383–386. 17251971 10.1038/nature05451

[9] Khan AI, Dinh DM, Schneider D, Lenski RE, Cooper TF (2011) Negative epistasis between beneficial mutations in an evolving bacterial population. Science 332: 1193–1196. 21636772 10.1126/science.1203801

[10] Chou HH, Chiu HC, Delaney NF, Segrè D, Marx CJ (2011) Diminishing returns epistasis among beneficial mutations decelerates adaptation. Science 332: 1190–1192. 21636771 10.1126/science.1203799 PMC3244271

[11] Sengupta AM, Djordjevic M, Shraiman BI (2002) Specificity and robustness in transcription control networks. Proc Natl Acad Sci USA 99: 2072–2077. 11854503 10.1073/pnas.022388499 PMC122321

[12] Gerland U, Hwa T (2002) On the selection and evolution of regulatory DNA motifs. J Mol Evol 55: 386–400. 12355260 10.1007/s00239-002-2335-z

[13] Berg J, Lässig M (2003) Stochastic evolution of transcription factor binding sites. Biophysics (Moscow) 48: S36–S44.

[14] Berg J, Willmann S, Lässig M (2004) Adaptive evolution of transcription factor binding sites. BMC Evol Biol 4: 42. 15511291 10.1186/1471-2148-4-42 PMC535555

[15] Bloom JD, et al (2005) Thermodynamic prediction of protein neutrality. Proc Natl Acad Sci USA 102: 606–611. 15644440 10.1073/pnas.0406744102 PMC545518

[16] DePristo MA, Weinreich DM, Hartl DL (2005) Missense meanderings in sequence space: a biophysical view of protein evolution. Nat Rev Genet 6: 678–687. 16074985 10.1038/nrg1672

[17] Bloom JD, Labthavikul ST, Otey CR, Arnold FH (2006) Protein stability promotes evolvability. Proc Natl Acad Sci USA 103: 5869–5874. 16581913 10.1073/pnas.0510098103 PMC1458665

[18] Zeldovich KB, Chen P, Shakhnovich EI (2007) Protein stability imposes limits on organism complexity and speed of molecular evolution. Proc Natl Acad Sci USA 104: 16152–16157. 17913881 10.1073/pnas.0705366104 PMC2042177

[19] Lässig M (2007) From biophysics to evolutionary genetics: statistical aspects of gene regulation. BMC Bioinformatics 8: S7. 17903288 10.1186/1471-2105-8-S6-S7 PMC1995540

[20] Bloom JD, Raval A, Wilke CO (2007) Thermodynamics of neutral protein evolution. Genetics 175: 255–266. 17110496 10.1534/genetics.106.061754 PMC1775007

[21] Bershtein S, Goldin K, Tawfik DS (2008) Intense neutral drifts yield robust and evolvable consensus proteins. J Mol Biol 379: 1029–1044. 18495157 10.1016/j.jmb.2008.04.024

[22] Mustonen V, Kinney J, Callan CG, Lässig M (2008) Energy-dependent fitness: A quantitative model for the evolution of yeast transcription factor binding sites. Proc Natl Acad Sci USA 105: 12376–12381. 18723669 10.1073/pnas.0805909105 PMC2527919

[23] Bloom JD, Glassman MJ (2009) Inferring stabilizing mutations from protein phylogenies: Application to influenza hemagglutinin. PLoS Comput Biol 5: e1000349. 19381264 10.1371/journal.pcbi.1000349 PMC2664478

[24] Manhart M, Morozov AV (2013) Path-based approach to random walks on networks characterizes how proteins evolve new functions. Phys Rev Lett 111: 088102. 24010480 10.1103/PhysRevLett.111.088102

[25] Manhart M, Morozov AV (2014) in First-Passage Phenomena and Their Applications, eds. Metzler R, Oshanin G, Redner S. (World Scientific, Singapore).

[26] Ptashne M, Gann A (2002) Genes and Signals. (Cold Spring Harbor Laboratory Press, Cold Spring Harbor).

[27] Rhee HS, Pugh BF (2011) Comprehensive genome-wide protein-DNA interactions detected at single-nucleotide resolution. Cell 147: 1408–1419. 22153082 10.1016/j.cell.2011.11.013 PMC3243364

[28] Lee TI, et al (2002) Transcriptional regulatory networks in Saccharomyces cerevisiae. Science 298: 799–804. 12399584 10.1126/science.1075090

[29] Harbison CT, et al (2004) Transcriptional regulatory code of a eukaryotic genome. Nature 431: 99–104. 15343339 10.1038/nature02800 PMC3006441

[30] MacIsaac K, et al (2006) An improved map of conserved regulatory sites for *Saccharomyces cerevisiae* . BMC Bioinformatics 7: 113. 16522208 10.1186/1471-2105-7-113 PMC1435934

[31] Chen K, van Nimwegen E, Rajewsky N, Siegal ML (2010) Correlating gene expression variation with cis-regulatory polymorphism in *Saccharomyces cerevisiae* . Genome Biol Evol 2: 697–707. 20829281 10.1093/gbe/evq054 PMC2953268

[32] Stormo GD, Fields DS (1998) Specificity, free energy and information content in protein-DNA interactions. TIBS 23: 109–113. 9581503 10.1016/s0968-0004(98)01187-6

[33] Berger MF, et al (2006) Compact, universal DNA microarrays to comprehensively determine transcription-factor binding site specificities. Nat Biotech 24: 1429–1435. 10.1038/nbt1246 PMC4419707 16998473

[34] Foat BC, Morozov AV, Bussemaker HJ (2006) Statistical mechanical modeling of genome-wide transcription factor occupancy data by MatrixREDUCE. Bioinformatics 22: e141–e149. 16873464 10.1093/bioinformatics/btl223

[35] Fordyce PM, et al (2010) De novo identification and biophysical characterization of transcription-factor binding sites with microfluidic affinity analysis. Nature Biotech 28: 970–975. 10.1038/nbt.1675 PMC2937095 20802496

[36] Kimura M (1983) The Neutral Theory of Molecular Evolution. (Cambridge University Press., Cambridge).

[37] Sella G, Hirsh AE (2005) The application of statistical physics to evolutionary biology. Proc Natl Acad Sci USA 102: 9541–9546. 15980155 10.1073/pnas.0501865102 PMC1172247

[38] Manhart M, Haldane A, Morozov AV (2012) A universal scaling law determines time reversibility and steady state of substitutions under selection. Theor Popul Biol 82: 66–76. 22838027 10.1016/j.tpb.2012.03.007 PMC3613437

[39] Winzeler EA, et al (1999) Functional characterization of the *S. cerevisiae* genome by gene deletion and parallel analysis. Science 285: 901–906. 10436161 10.1126/science.285.5429.901

[40] Berg OG, von Hippel PH (1987) Selection of DNA binding sites by regulatory proteins: Statistical-mechanical theory and application to operators and promoters. J Mol Biol 193: 723–743. 3612791 10.1016/0022-2836(87)90354-8

[41] Champagnat N (2006) A microscopic interpretation for adaptive dynamics trait substitution sequence models. Stoch Proc Appl 116: 1127–1160.

[42] Kimura M, Ohta T (1969) The average number of generations until fixation of a mutant gene in a finite population. Genetics 61: 763–771. 17248440 10.1093/genetics/61.3.763 PMC1212239

[43] Ewens W (2004) Mathematical Population Genetics. (Springer, New York).

[44] Wakeley J (2005) The limits of theoretical population genetics. Genetics 169: 1–7. 15677744 10.1093/genetics/169.1.1 PMC1448894

[45] Tsai IJ, Bensasson D, Burt A, Koufopanou V (2008) Population genomics of the wild yeast *Saccharomyces paradoxus*: Quantifying the life cycle. Proc Natl Acad Sci USA 105: 4957–4962. 18344325 10.1073/pnas.0707314105 PMC2290798

[46] Dujon B (2010) Yeast evolutionary genomics. Nat Rev Genet 11: 512–524. 20559329 10.1038/nrg2811

[47] Liti G, et al (2009) Population genomics of domestic and wild yeasts. Nature 458: 337–341. 19212322 10.1038/nature07743 PMC2659681

[48] Doniger SW, et al (2008) A catalog of neutral and deleterious polymorphism in yeast. PLoS Genet 4: e1000183. 18769710 10.1371/journal.pgen.1000183 PMC2515631

[49] Fay JC, Benavides JA (2005) Evidence for domesticated and wild populations of *Saccharomyces cerevisiae* . PLoS Genet 1: 66–71. 16103919 10.1371/journal.pgen.0010005 PMC1183524

[50] Replansky T, Koufopanou V, Greig D, Bell G (2008) *Saccharomyces sensu stricto* as a model system for evolution and ecology. Trends Ecol Evol (Amst) 23: 494–501. 18656281 10.1016/j.tree.2008.05.005

[51] Giaever G, et al (2002) Functional profiling of the *Saccharomyces cerevisiae* genome. Nature 418: 387–391. 12140549 10.1038/nature00935

[52] Holstege FCP, et al (1998) Dissecting the regulatory circuitry of a eukaryotic genome. Cell 95: 717–728. 9845373 10.1016/s0092-8674(00)81641-4

[53] Hahn MW, Stajich JE, Wray GA (2003) The effects of selection against spurious transcription factor binding sites. Mol Biol Evol 20: 901–906. 12716998 10.1093/molbev/msg096

[54] Djordjevic M, Sengupta AM, Shraiman BI (2003) A biophysical approach to transcription factor binding site discovery. Genome Res 13: 2381–2390. 14597652 10.1101/gr.1271603 PMC403756

[55] Burnham KP, Anderson DR (2002) Model Selection and Multimodal Inference: A Practical Information-Theoretic Approach. (Springer-Verlag, New York), Second edition.

[56] Jordan IK, Rogozin IB, Wolf YI, Koonin EV (2002) Essential genes are more evolutionarily conserved than are nonessential genes in bacteria. Genome Res 12: 962–968. 12045149 10.1101/gr.87702 PMC1383730

[57] Pal C, Papp B, Hurst LD (2003) Genomic function (communication arising): Rate of evolution and gene dispensability. Nature 421: 496–497. 12556881 10.1038/421496b

[58] Zhang J, He X (2005) Significant impact of protein dispensability on the instantaneous rate of protein evolution. Mol Biol Evol 22: 1147–1155. 15689524 10.1093/molbev/msi101

[59] Choi JK, Kim SC, Seo J, Kim S, Bhak J (2007) Impact of transcriptional properties on essentiality and evolutionary rate. Genetics 175: 199–206. 17057246 10.1534/genetics.106.066027 PMC1775009

[60] Krylov DM, Wolf YI, Rogozin IB, Koonin EV (2003) Gene loss, protein sequence divergence, gene dispensability, expression level, and interactivity are correlated in eukaryotic evolution. Genome Res 13: 2229–2235. 14525925 10.1101/gr.1589103 PMC403683

[61] Wang Z, Zhang J (2009) Why is the correlation between gene importance and gene evolutionary rate so weak? PLoS Genet 5: e1000329. 19132081 10.1371/journal.pgen.1000329 PMC2605560

[62] Fang G, Rocha E, Danchin A (2005) How essential are nonessential genes? Mol Biol Evol 22: 2147–2156. 16014871 10.1093/molbev/msi211

[63] Hirsh AE, Fraser HB (2001) Protein dispensability and rate of evolution. Nature 411: 1046–1049. 11429604 10.1038/35082561

[64] Doniger SW, Fay JC (2007) Frequent gain and loss of functional transcription factor binding sites. PLoS Comput Biol 3: e99. 17530920 10.1371/journal.pcbi.0030099 PMC1876492

[65] Raijman D, Shamir R, Tanay A (2008) Evolution and selection in yeast promoters: Analyzing the combined effect of diverse transcription factor binding sites. PLoS Comput Biol 4: e7. 18193940 10.1371/journal.pcbi.0040007 PMC2186363

[66] Tirosh I, Weinberger A, Bezalel D, Kaganovich M, Barkai N (2008) On the relation between promoter divergence and gene expression evolution. Mol Syst Biol 4. 10.1038/msb4100198 PMC2238714 18197176

[67] Tuch BB, Galgoczy DJ, Hernday AD, Li H, Johnson AD (2008) The evolution of combinatorial gene regulation in fungi. PLoS Biol 6: e38. 18303948 10.1371/journal.pbio.0060038 PMC2253631

[68] Jovelin R, Phillips P (2009) Evolutionary rates and centrality in the yeast gene regulatory network. Genome Biol 10: R35. 19358738 10.1186/gb-2009-10-4-r35 PMC2688926

[69] Wuchty S, Oltvai ZN, Barabasi AL (2003) Evolutionary conservation of motif constituents in the yeast protein interaction network. Nat Genet 35: 176–179. 12973352 10.1038/ng1242

[70] van Dijk ADJ, van Mourik S, van Ham RCHJ (2012) Mutational robustness of gene regulatory networks. PLoS ONE 7: e30591. 22295094 10.1371/journal.pone.0030591 PMC3266278

[71] He X, Duque TSPC, Sinha S (2012) Evolutionary origins of transcription factor binding site clusters. Mol Biol Evol 29: 1059–1070. 22075113 10.1093/molbev/msr277 PMC3278477

[72] He BZ, Holloway AK, Maerkl SJ, Kreitman M (2011) Does positive selection drive transcription factor binding site turnover? A test with *Drosophila* cis-regulatory modules. PLoS Genet 7: e1002053. 21572512 10.1371/journal.pgen.1002053 PMC3084208

[73] Habib N, Wapinski I, Margalit H, Regev A, Friedman N (2012) A functional selection model explains evolutionary robustness despite plasticity in regulatory networks. Mol Syst Biol 8. 10.1038/msb.2012.50 PMC3501536 23089682

[74] Allen LJS (2011) An Introduction to Stochastic Processes with Applications to Biology. (Chapman and Hall, CRC, Boca Raton), Second edition.

[75] Cherry JM, et al (2012) *Saccharomyces* Genome Database: the genomics resource of budding yeast. Nucl Acids Res 40: D700–D705. 22110037 10.1093/nar/gkr1029 PMC3245034

[76] Larkin MA, et al (2007) Clustal W and Clustal X version 2.0. Bioinformatics 23: 2947–2948. 17846036 10.1093/bioinformatics/btm404

[77] Yang Z (2007) PAML 4: Phylogenetic Analysis by Maximum Likelihood. Mol Biol Evol 24: 1586–1591. 17483113 10.1093/molbev/msm088

