# Supplementary material for: Biophysical Fitness Landscapes for Transcription Factor Binding Sites
Source: PLoS Comput Biol. 2014 Jul 10;10(7):e1003683. doi: 10.1371/journal.pcbi.1003683 (PMC4091707; doi:10.1371/journal.pcbi.1003683)

(A) Dataset | **(B) Growth Rate** | **(C) Expression Level** | **(D) K_A/K_S Ratio** | **(E) TSS Distance**

REB1 (essential TF)

Total sites: 749
Unique sites: 235

	**Essential**	**Noness.**	p
Total Data	213	468	
Expr Data	209	433	
S. Par. Data	194	358	
$\langle E \rangle$	-12.632	-12.540	0.090
V	0.336	0.471	0.245
$\langle \Delta E^2 \rangle$	0.103	0.206	0.152
$\langle d \rangle$	0.847	0.904	0.511

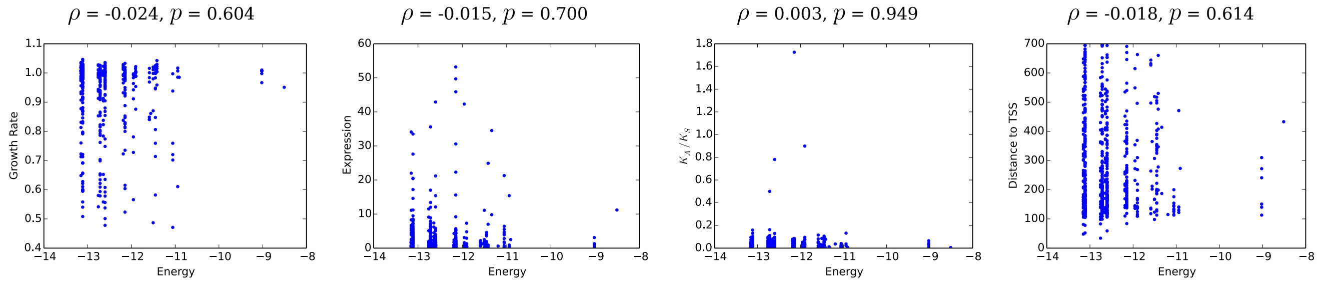

ROX1 (nonessential TF)

Total sites: 93
Unique sites: 58

	**Essential**	**Noness.**	p
Total Data	18	65	
Expr Data	18	64	
S. Par. Data	18	47	
$\langle E \rangle$	-11.683	-11.575	0.680
V	0.205	0.833	0.546
$\langle \Delta E^2 \rangle$	0.015	0.697	0.274
$\langle d \rangle$	0.824	0.737	0.779

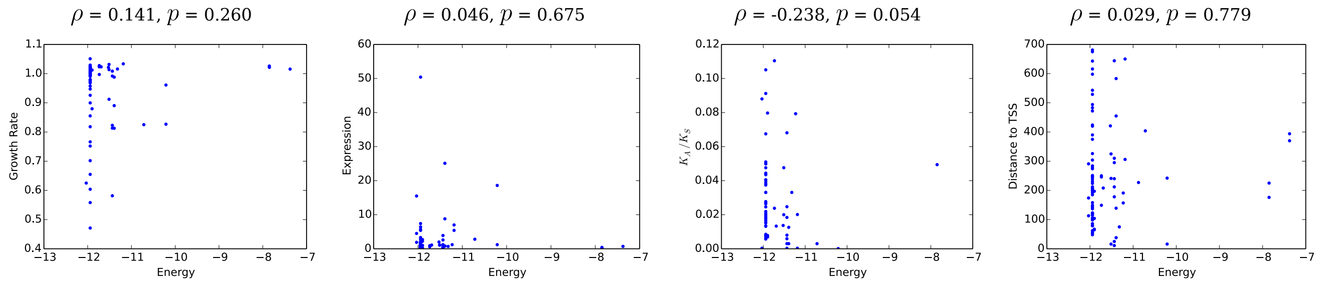

MET32 (nonessential TF)

Total sites: 68
Unique sites: 39

	**Essential**	**Noness.**	p
Total Data	5	59	
Expr Data	4	49	
S. Par. Data	4	48	
$\langle E \rangle$	-8.660	-9.738	0.055
V	3.917	1.136	0.010
$\langle \Delta E^2 \rangle$	0.000	0.006	1.000
$\langle d \rangle$	0.250	0.377	1.000

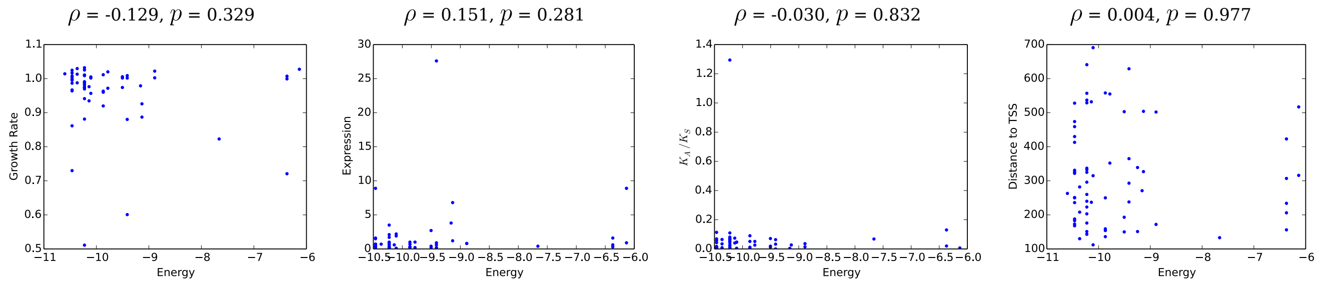

RPN4 (nonessential TF)

Total sites: 188
Unique sites: 38

	**Essential**	**Noness.**	p
Total Data	71	101	
Expr Data	71	89	
S. Par. Data	66	77	
$\langle E \rangle$	-10.003	-9.776	0.048
V	0.304	0.705	0.039
$\langle \Delta E^2 \rangle$	0.084	0.126	0.707
$\langle d \rangle$	0.167	0.231	0.441

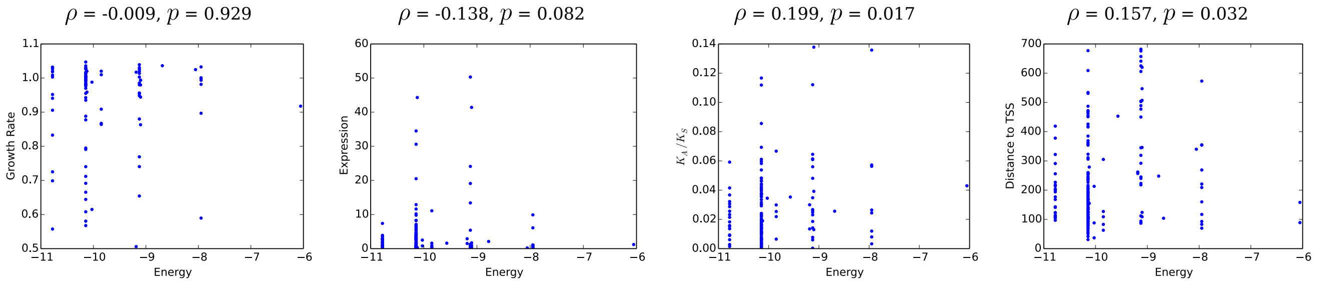

MET31 (nonessential TF)

Total sites: 77
Unique sites: 35

	**Essential**	**Noness.**	p
Total Data	10	60	
Expr Data	9	53	
S. Par. Data	10	48	
$\langle E \rangle$	-9.968	-10.201	0.391
V	0.328	0.649	0.506
$\langle \Delta E^2 \rangle$	0.000	0.029	0.580
$\langle d \rangle$	0.000	0.151	0.345

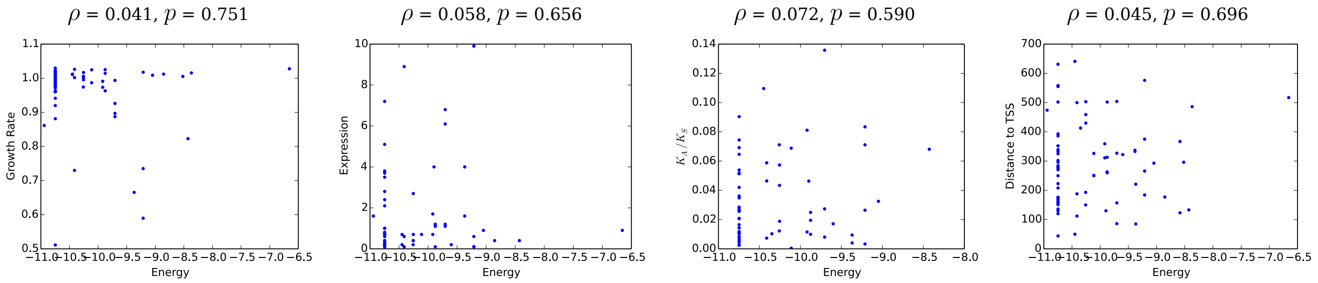

PDR3 (nonessential TF)

Total sites: 73
Unique sites: 31

	**Essential**	**Noness.**	p
Total Data	6	51	
Expr Data	6	47	
S. Par. Data	5	36	
$\langle E \rangle$	-7.340	-7.942	0.173
V	1.826	0.968	0.441
$\langle \Delta E^2 \rangle$	3.300	0.121	0.014
$\langle d \rangle$	1.000	0.304	0.033

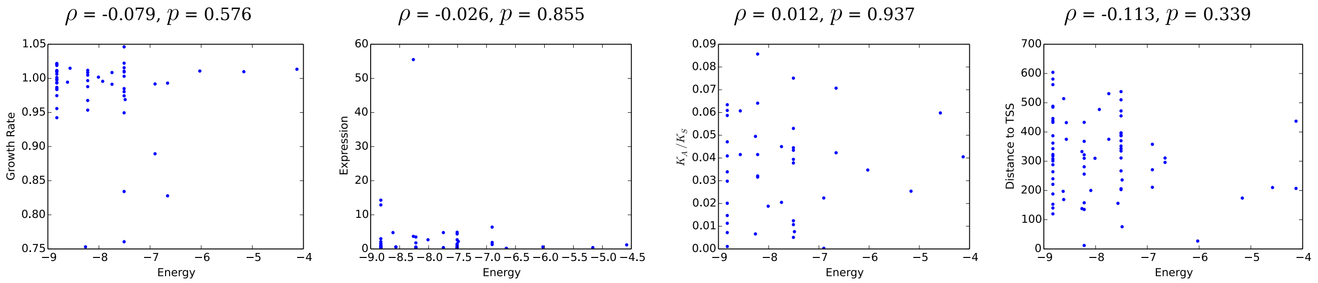

YAP7 (nonessential TF)

Total sites: 36
Unique sites: 22

	Essential	Noness.	p
Total Data	13	23	
Expr Data	13	23	
S. Par. Data	11	18	
$\langle E \rangle$	-9.076	-9.936	0.158
V	2.575	2.865	0.828
$\langle \Delta E^2 \rangle$	1.718	1.073	0.717
$\langle d \rangle$	0.583	0.182	0.093

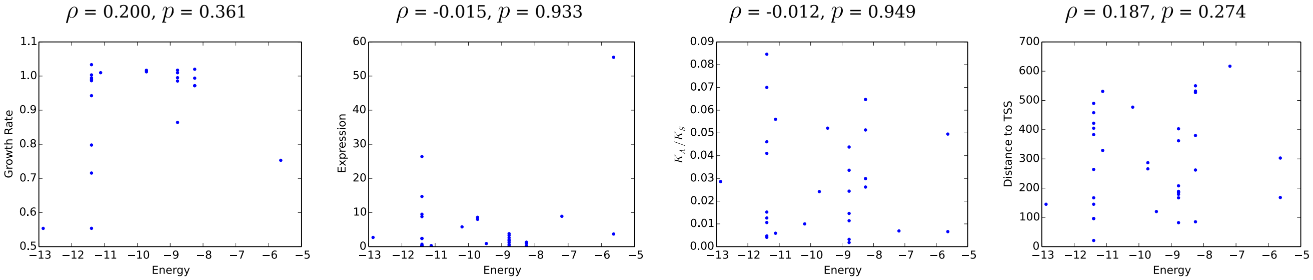

BAS1 (nonessential TF)

Total sites: 41
Unique sites: 21

	Essential	Noness.	p
Total Data	5	30	
Expr Data	5	26	
S. Par. Data	5	28	
$\langle E \rangle$	-12.107	-11.518	0.589
V	0.322	5.328	0.050
$\langle \Delta E^2 \rangle$	0.128	0.600	0.968
$\langle d \rangle$	0.200	0.214	1.000

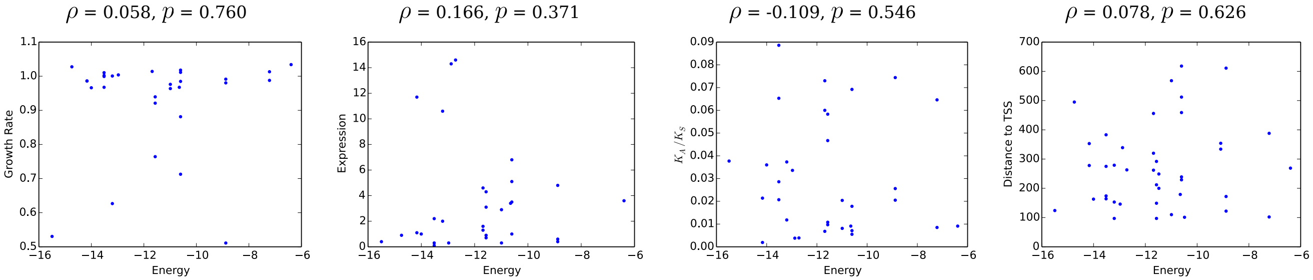

STB5 (nonessential TF)

Total sites: 28
Unique sites: 19

	Essential	Noness.	p
Total Data	5	20	
Expr Data	5	18	
S. Par. Data	5	14	
$\langle E \rangle$	-9.893	-9.918	0.912
V	0.317	0.116	0.074
$\langle \Delta E^2 \rangle$	0.002	0.000	0.217
$\langle d \rangle$	0.400	0.222	0.636

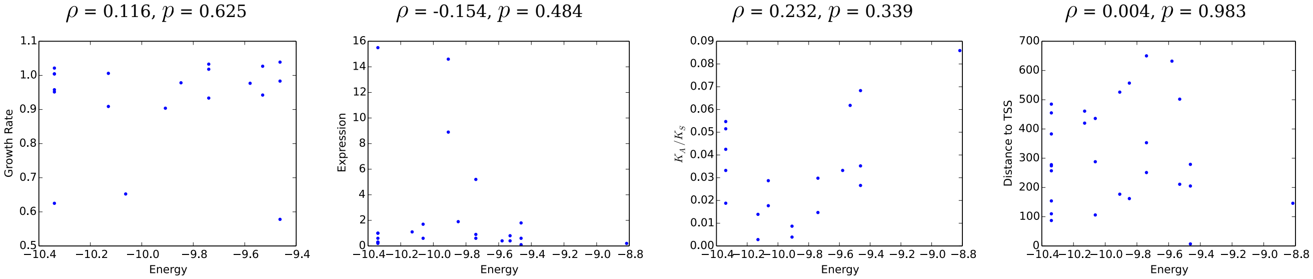

AFT1 (nonessential TF)

Total sites: 42
Unique sites: 18

	**Essential**	**Noness.**	p
Total Data	5	30	
Expr Data	5	29	
S. Par. Data	5	23	
$\langle E \rangle$	-11.475	-11.425	0.603
V	0.006	0.037	0.128
$\langle \Delta E^2 \rangle$	0.000	0.018	0.673
$\langle d \rangle$	0.400	0.391	1.000

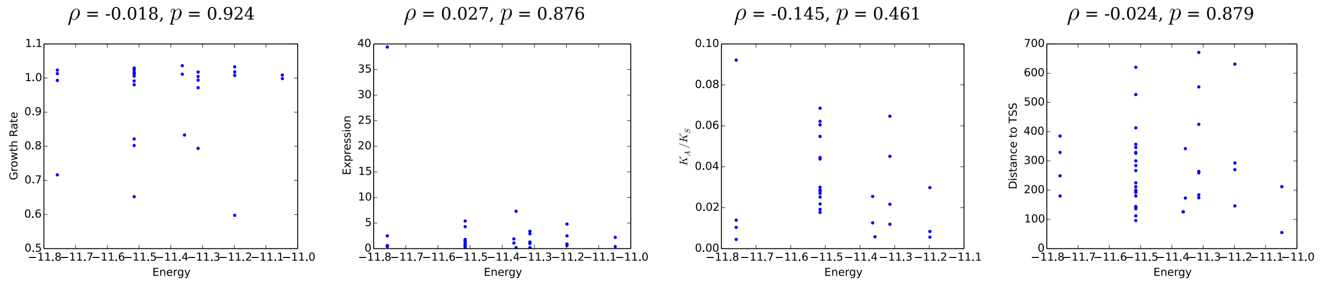

CUP9 (nonessential TF)

Total sites: 58
Unique sites: 13

	**Essential**	**Noness.**	p
Total Data	11	43	
Expr Data	11	32	
S. Par. Data	11	31	
$\langle E \rangle$	-11.681	-11.607	0.753
V	0.141	0.494	0.290
$\langle \Delta E^2 \rangle$	0.024	0.109	0.907
$\langle d \rangle$	0.100	0.139	1.000

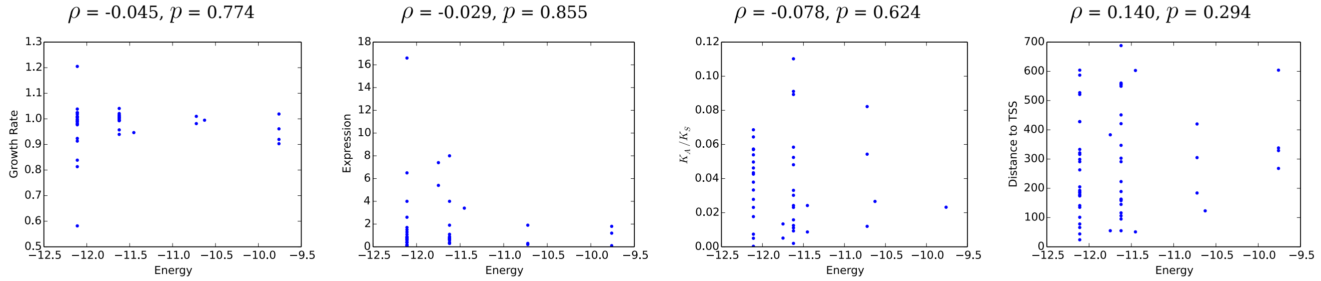

MCM1 (essential TF)

Total sites: 18
Unique sites: 13

	**Essential**	**Noness.**	p
Total Data	2	15	
Expr Data	2	12	
S. Par. Data	2	12	
$\langle E \rangle$	-9.252	-8.580	0.794
V	0.000	9.927	0.080
$\langle \Delta E^2 \rangle$	0.013	1.360	0.882
$\langle d \rangle$	2.000	0.800	0.132

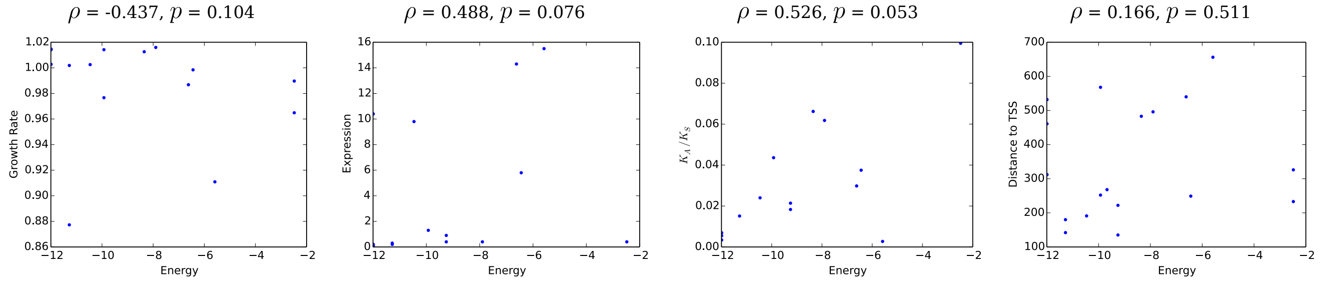

CIN5 (nonessential TF)

Total sites: 19
Unique sites: 12

	**Essential**	**Noness.**	p
Total Data	2	13	
Expr Data	2	9	
S. Par. Data	2	11	
$\langle E \rangle$	-13.841	-13.625	0.886
V	0.046	1.290	1.000
$\langle \Delta E^2 \rangle$	0.030	0.036	1.000
$\langle d \rangle$	1.000	0.417	0.461

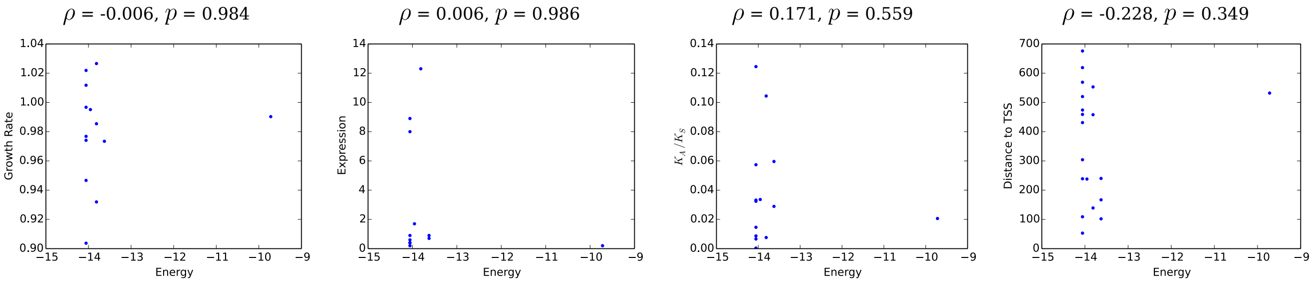

GAT1 (nonessential TF)

Total sites: 88
Unique sites: 11

	**Essential**	**Noness.**	p
Total Data	8	70	
Expr Data	8	63	
S. Par. Data	7	60	
$\langle E \rangle$	-10.036	-10.046	0.944
V	0.041	0.035	0.543
$\langle \Delta E^2 \rangle$	0.027	0.017	1.000
$\langle d \rangle$	0.429	0.353	1.000

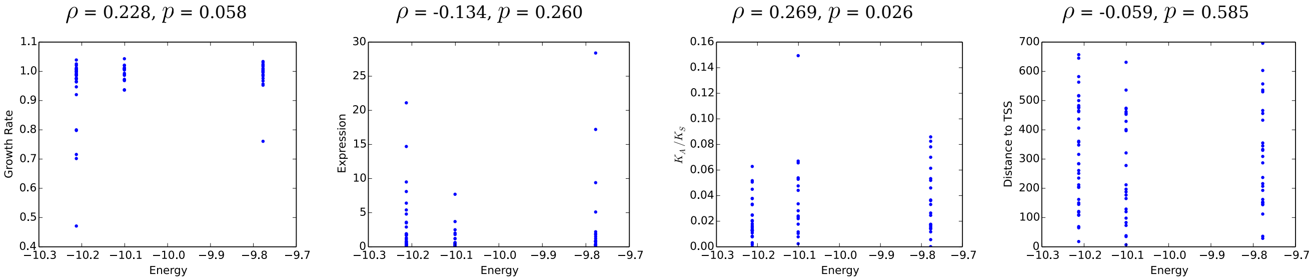

MSN2 (nonessential TF)

Total sites: 141
Unique sites: 8

	**Essential**	**Noness.**	p
Total Data	19	106	
Expr Data	18	96	
S. Par. Data	15	86	
$\langle E \rangle$	-8.433	-8.204	0.662
V	1.577	2.251	0.735
$\langle \Delta E^2 \rangle$	0.985	0.664	1.000
$\langle d \rangle$	0.059	0.079	1.000

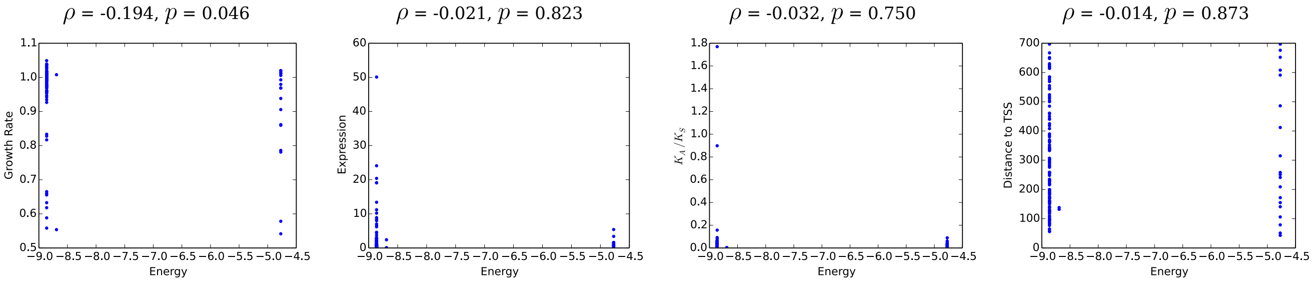

CAD1 (nonessential TF)

Total sites: 28
Unique sites: 8

	**Essential**	**Noness.**	p
Total Data	3	25	
Expr Data	3	25	
S. Par. Data	3	20	
$\langle E \rangle$	-7.910	-8.635	0.465
V	3.585	1.571	0.174
$\langle \Delta E^2 \rangle$	0.000	0.301	0.932
$\langle d \rangle$	0.000	0.200	1.000

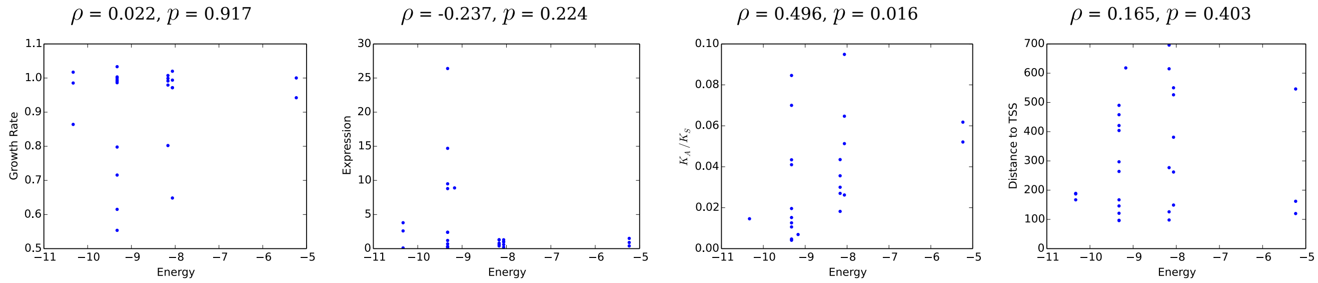

ACE2 (nonessential TF)

Total sites: 45
Unique sites: 6

	**Essential**	**Noness.**	p
Total Data	7	29	
Expr Data	7	26	
S. Par. Data	6	23	
$\langle E \rangle$	-11.023	-10.954	0.552
V	0.094	0.065	0.374
$\langle \Delta E^2 \rangle$	0.000	0.000	1.000
$\langle d \rangle$	0.000	0.000	1.000

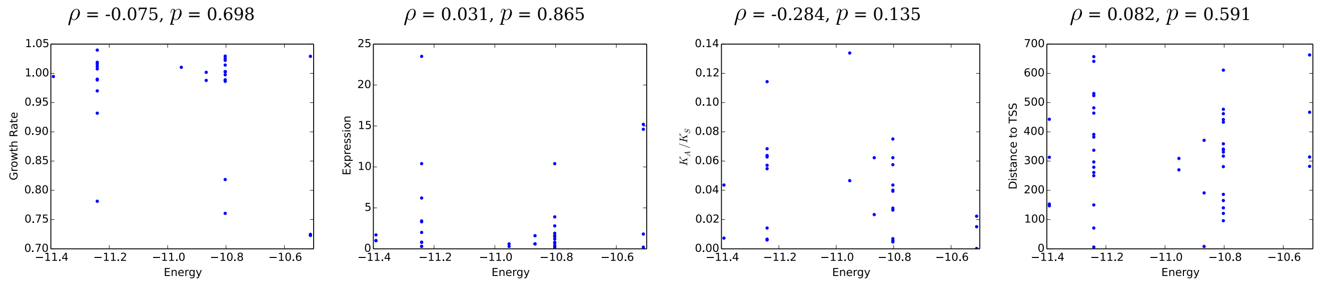

YAP3 (nonessential TF)

Total sites: 38
Unique sites: 6

	**Essential**	**Noness.**	p
Total Data	8	30	
Expr Data	8	25	
S. Par. Data	8	24	
$\langle E \rangle$	-13.718	-13.503	0.530
V	0.199	0.535	0.521
$\langle \Delta E^2 \rangle$	0.000	0.308	0.560
$\langle d \rangle$	0.000	0.276	0.172

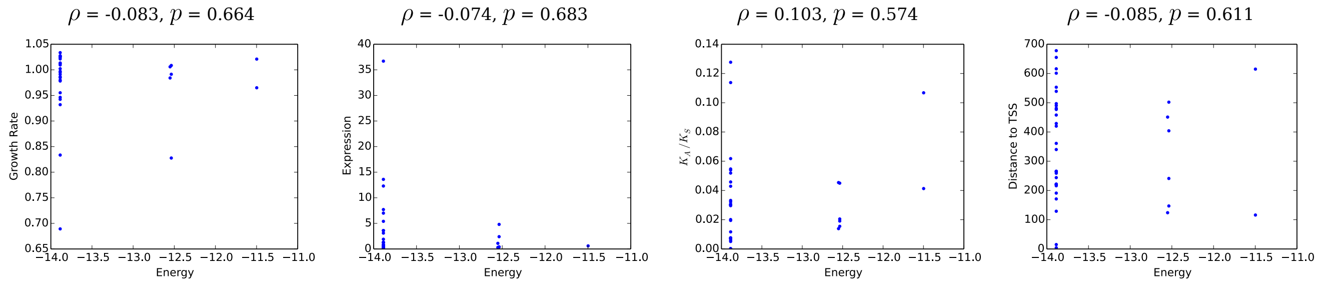

GCN4 (nonessential TF)

Total sites: 9
Unique sites: 5

	**Essential**	**Noness.**	p
Total Data	1	8	
Expr Data	1	7	
S. Par. Data	1	8	
$\langle E \rangle$	-14.357	-16.442	0.222
V	0.000	1.736	0.889
$\langle \Delta E^2 \rangle$	1.588	0.025	0.125
$\langle d \rangle$	2.000	0.429	0.125

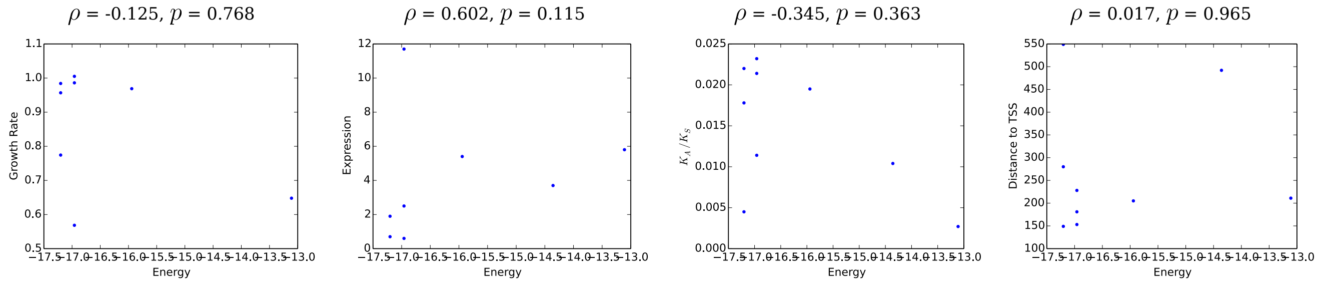

MATA2 (nonessential TF)

Total sites: 13
Unique sites: 4

	**Essential**	**Noness.**	p
Total Data	1	9	
Expr Data	1	9	
S. Par. Data	1	6	
$\langle E \rangle$	-8.465	-8.441	1.000
V	0.000	0.002	0.801
$\langle \Delta E^2 \rangle$	0.011	0.006	1.000
$\langle d \rangle$	1.000	0.500	1.000

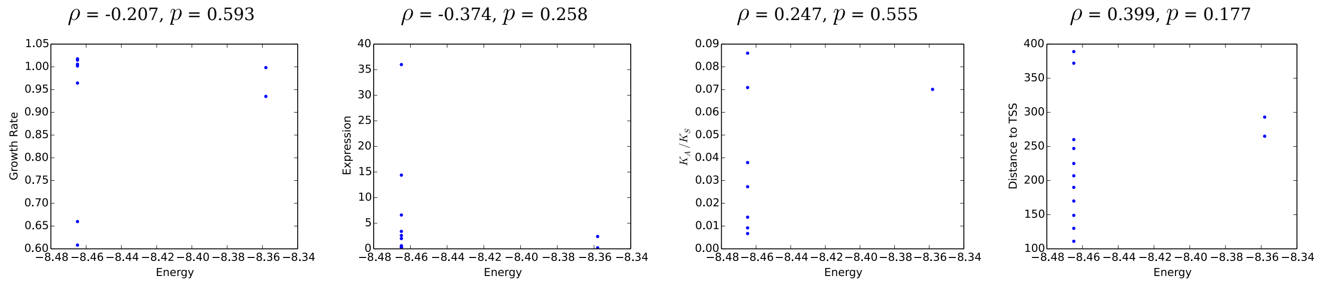

YAP1 (nonessential TF)

Total sites: 6
Unique sites: 4

	**Essential**	**Noness.**	p
Total Data	0	6	
Expr Data	0	6	
S. Par. Data	0	5	
$\langle E \rangle$	—	-9.143	—
V	—	2.114	—
$\langle \Delta E^2 \rangle$	—	3.349	—
$\langle d \rangle$	—	0.600	—

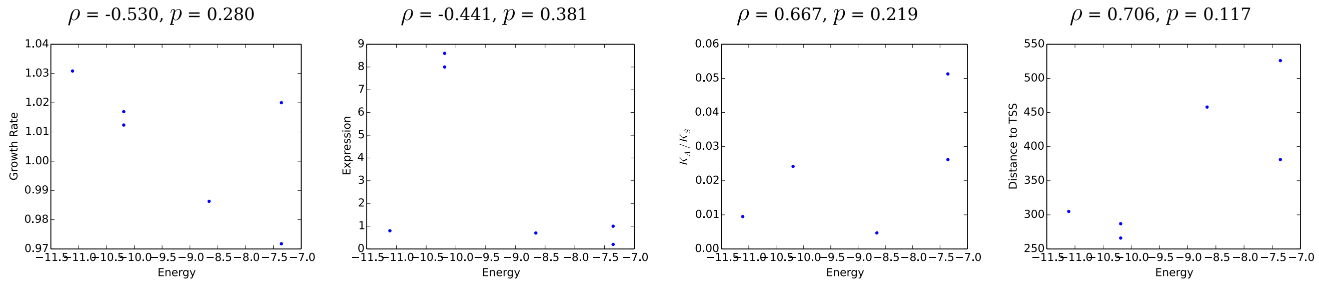

CBF1 (nonessential TF)

Total sites: 49
Unique sites: 3

	**Essential**	**Noness.**	p
Total Data	7	38	
Expr Data	7	38	
S. Par. Data	6	31	
$\langle E \rangle$	-8.060	-8.048	0.685
V	0.001	0.002	0.872
$\langle \Delta E^2 \rangle$	0.009	0.007	0.987
$\langle d \rangle$	0.143	0.139	1.000

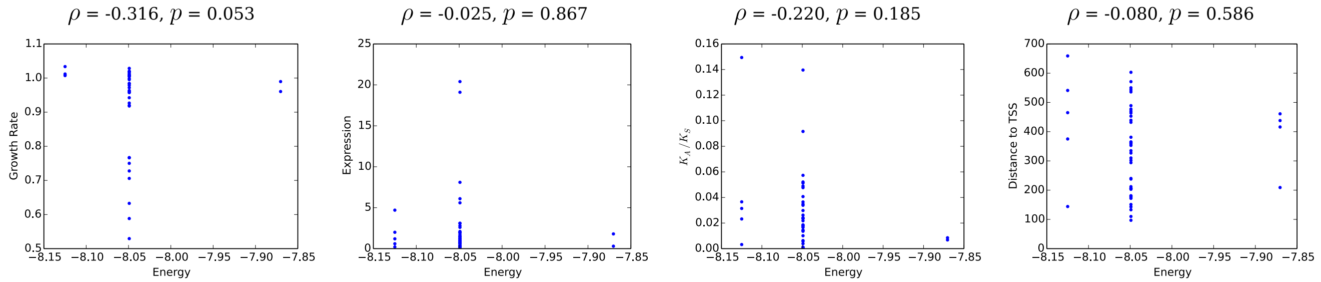

DAL80 (nonessential TF)

Total sites: 44
Unique sites: 3

	**Essential**	**Noness.**	p
Total Data	4	34	
Expr Data	4	31	
S. Par. Data	4	30	
$\langle E \rangle$	-11.245	-10.961	0.426
V	0.000	0.205	0.238
$\langle \Delta E^2 \rangle$	0.000	0.224	0.546
$\langle d \rangle$	0.000	0.303	0.545

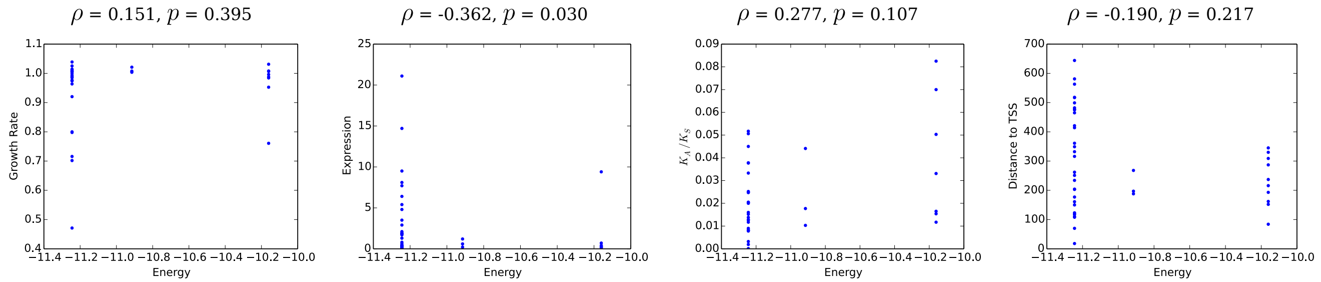

AFT2 (nonessential TF)

Total sites: 118
Unique sites: 2

	**Essential**	**Noness.**	p
Total Data	17	80	
Expr Data	17	71	
S. Par. Data	15	62	
$\langle E \rangle$	-13.505	-13.447	0.100
V	0.010	0.016	0.067
$\langle \Delta E^2 \rangle$	0.000	0.006	0.344
$\langle d \rangle$	0.000	0.087	0.343

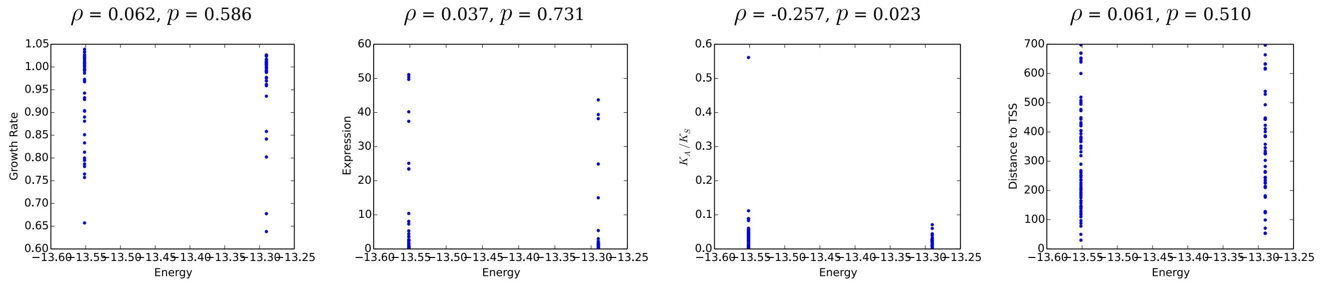

SKO1 (essential TF)

Total sites: 12
Unique sites: 2

	**Essential**	**Noness.**	p
Total Data	1	11	
Expr Data	1	10	
S. Par. Data	1	9	
$\langle E \rangle$	-7.801	-7.525	1.000
V	0.000	0.343	0.834
$\langle \Delta E^2 \rangle$	0.000	0.000	1.000
$\langle d \rangle$	0.000	0.000	1.000

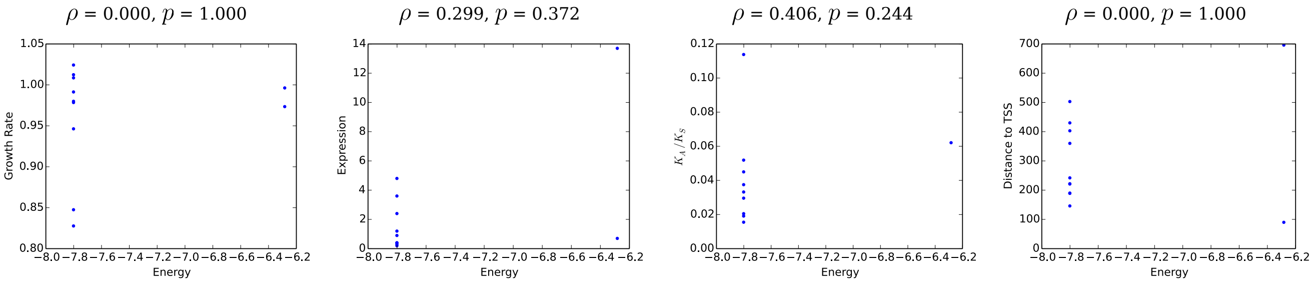

Supplement: Table S1 — Full summary of tests for site-specific selection. For 25 TFs we compute TF-DNA interaction energies (in kcal/mol) for each site. Columns from left to right: (A) Essentiality of the TF according to the Yeast Deletion Database; total number of binding sites for each TF; total number of sites with unique sequences. The table lists how many essential and nonessential genes are regulated by each TF, and how many of these genes have gene expression and S. paradoxus ratio data. We also report the mean energy and the variance of sites regulating both essential and nonessential genes, and mean squared energy difference and mean Hamming distance between S. cerevisiae and S. paradoxus sites regulating essential and nonessential genes. We show -values for the significance of the difference between these two classes of sites (see Methods). (B) Growth rate in strains with nonessential gene knockouts versus energy of TF binding sites regulating the knockout genes. (C) Gene expression versus energy of TF sites regulating the genes. (D) Ratio of nonsynonymous to synonymous substitutions () in genes versus energy of their TF regulatory sites. (E) Distance between each binding site and the closest transcription start site (TSS) versus the energy of the site. For (B)–(E) we report the Spearman rank correlation between each property and site energy, along with the -value of its significance (see Methods). (PDF) [file pcbi.1003683.s001.pdf]
