# Supplementary figures and images for: Biophysical Fitness Landscapes for Transcription Factor Binding Sites
Source: PLoS Comput Biol. 2014 Jul 10;10(7):e1003683. doi: 10.1371/journal.pcbi.1003683 (PMC4091707; doi:10.1371/journal.pcbi.1003683)

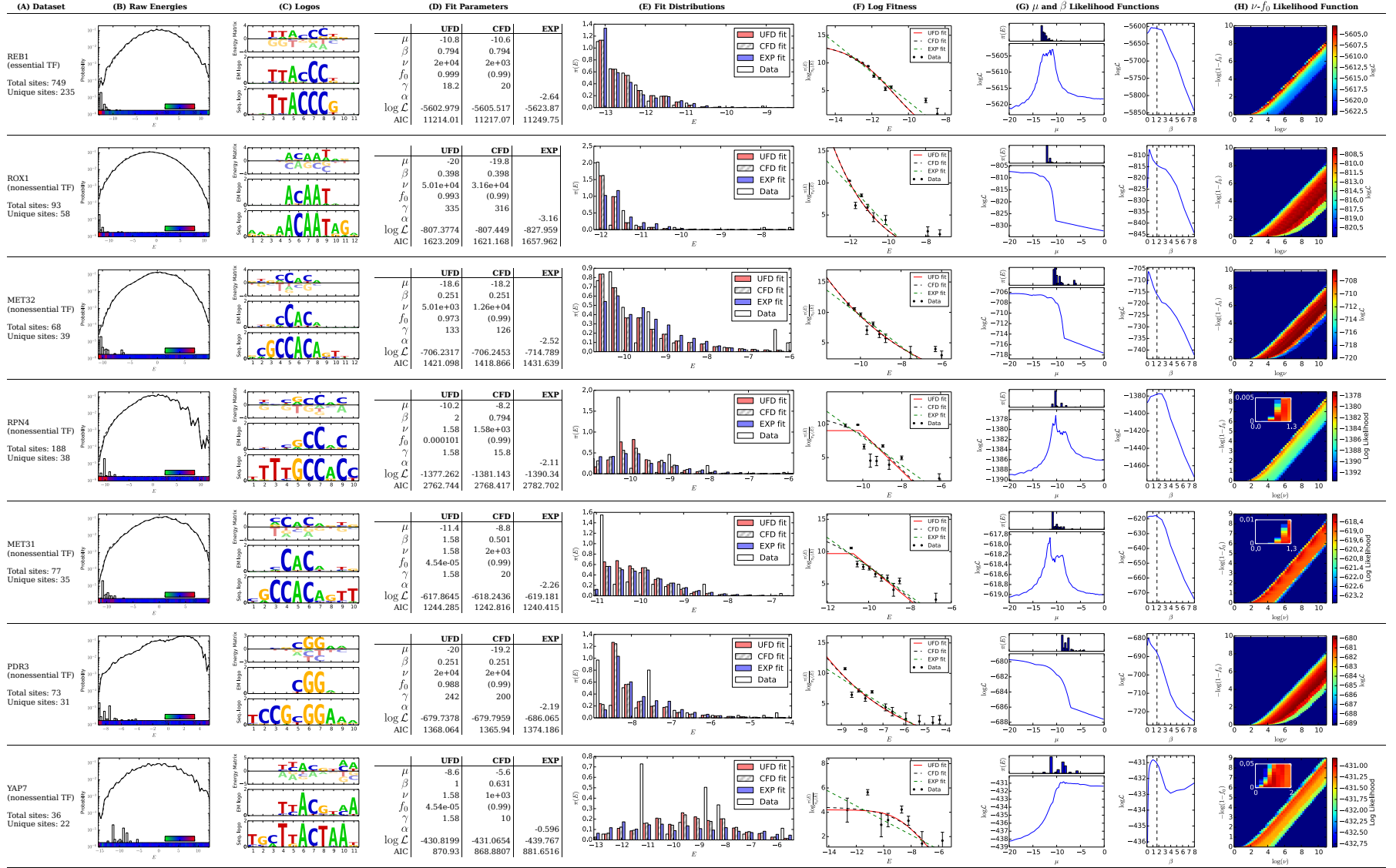

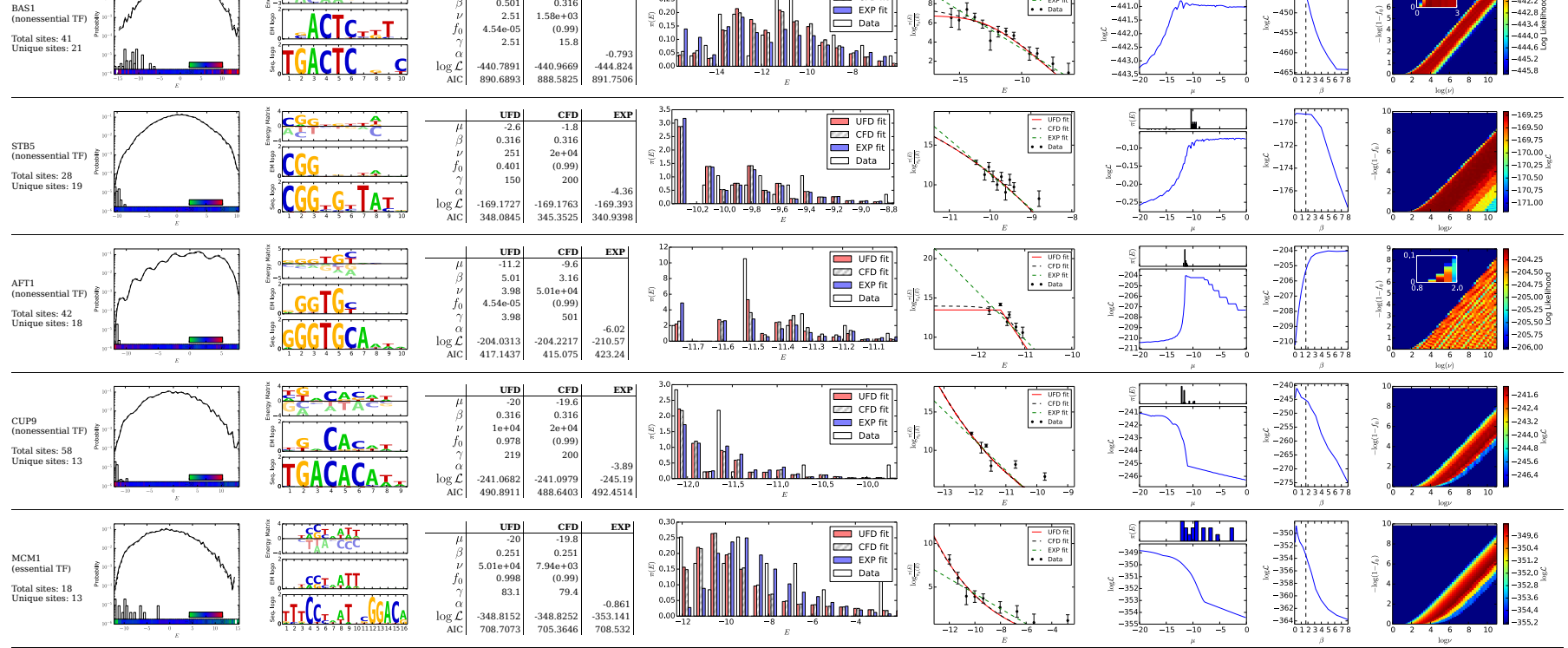

Supplement: Table S2 — Summary of fitness landscape fits to TF binding site data. We consider 12 TFs which have at least unique binding site sequences. Each row corresponds to a TF, ranked in the decreasing order of the number of unique binding site sequences. Columns, from left to right: (A) Summary of TF binding site data. (B) Same as Fig. 5A. (C) Same as Fig. 5B. (D) Fitted values of fitness landscape parameters, maximized log-likelihoods, and AICs for the unconstrained fit to the Fermi-Dirac function of Eq. 6 (“UFD”), constrained fit to the Eq. 6 with (“CFD”), and fit to an exponential fitness function (“EXP”). (E) Same as Fig. 5C. (F) Same as Fig. 5D. (G) Left panel: Log-likelihood of the unconstrained Fermi-Dirac model as a function of the effective chemical potential . For reference, the distribution of functional binding site energies (same as in (B)) is shown on top. Right panel: Log-likelihood as a function of the effective inverse temperature For reference, the inverse room temperature is shown as the vertical dashed line. To generate the log-likelihood plots, or were scanned across a range of values while all the other parameters were re-optimized for each new value of or . (H) Heatmap of log-likelihood as a function of and (note that constant corresponds to a straight line with slope 1 in these coordinates). For likelihoods that have a maximum near , insets show a zoomed-in view. To generate the log-likelihood heatmaps, and were scanned across the region shown while the other parameters ( and ) were re-optimized at each point separately. (PDF) [file pcbi.1003683.s002.pdf]
