# Supplementary material for: Biophysical Fitness Landscapes for Transcription Factor Binding Sites
Source: PLoS Comput Biol. 2014 Jul 10;10(7):e1003683. doi: 10.1371/journal.pcbi.1003683 (PMC4091707; doi:10.1371/journal.pcbi.1003683)

(A) Hessian Eigenvectors **(B) Subsample Fits**

REB1

λ	$\log\nu$	μ	$\log\beta$	$\mathcal{T}(f_0)$
-0.0201	0.894	0.001	-0	0.447
-102	-0.097	-0.81	0.545	0.195
-179	0.062	-0.578	-0.804	-0.122
-1.29e+04	-0.432	0.101	-0.237	0.864

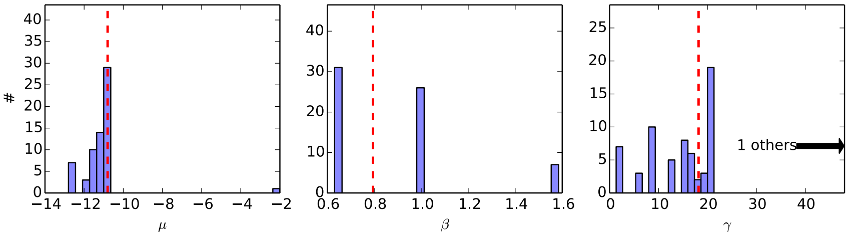

ROX1

λ	$\log\nu$	μ	$\log\beta$	$\mathcal{T}(f_0)$
0.00498	0.891	0.04	0	0.452
-0.0333	-0.1	0.989	0.008	0.111
-34.8	0.42	0.134	0.321	-0.838
-2.32e+04	0.141	0.054	-0.947	-0.283

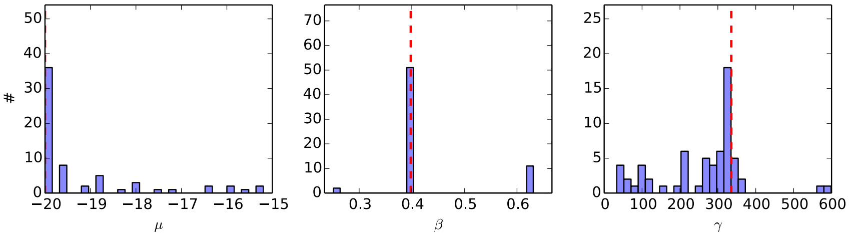

MET32

λ	$\log\nu$	μ	$\log\beta$	$\mathcal{T}(f_0)$
0.00356	0.885	0.1	0	0.454
-0.00198	-0.126	0.992	0.006	0.027
-169	0.403	0.071	0.436	-0.802
-1.46e+04	0.194	0.041	-0.9	-0.388

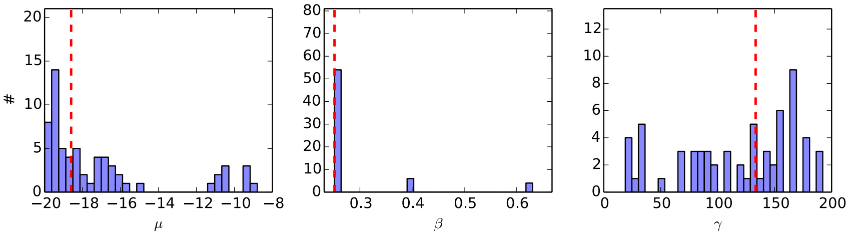

RPN4

λ	$\log\nu$	μ	$\log\beta$	$\mathcal{T}(f_0)$
0.938	-0.235	-0.007	0.42	-0.877
-7.09	0.265	-0.273	-0.805	-0.454
-58.9	0.371	0.91	-0.1	-0.155
-705	0.859	-0.311	0.406	-0.033

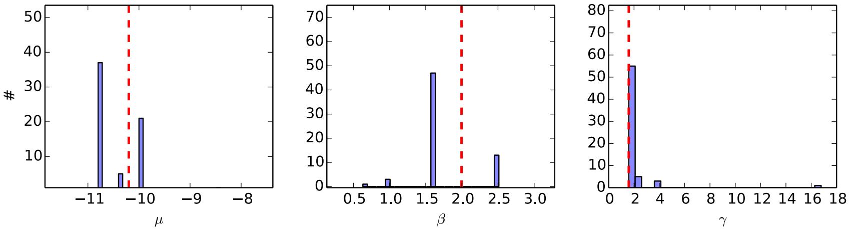

MET31

λ	$\log\nu$	μ	$\log\beta$	$\mathcal{T}(f_0)$
-0.375	-0.186	0.052	0.254	-0.948
-3.23	0.132	-0.893	-0.39	-0.179
-26.5	-0.501	-0.433	0.702	0.263
-358	0.835	-0.107	0.539	-0.025

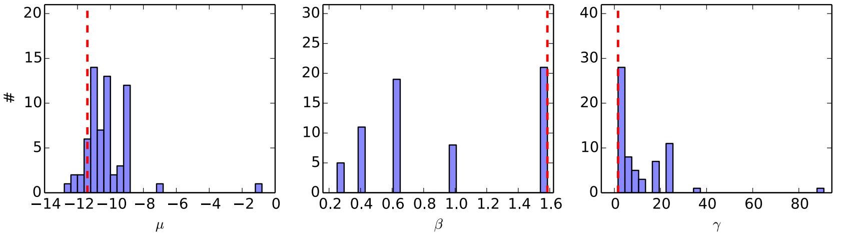

PDR3

λ	$\log\nu$	μ	$\log\beta$	$\mathcal{T}(f_0)$
0.00138	0.889	0.066	-0	0.453
-0.0051	-0.107	0.992	-0.001	0.066
-20.7	0.43	0.104	0.262	-0.858
-2.78e+04	0.117	0.027	-0.965	-0.233

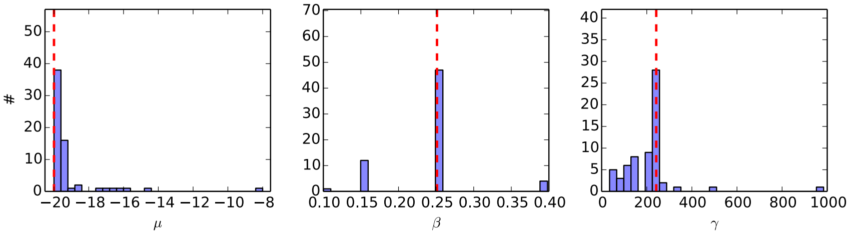

YAP7

λ	$\log\nu$	μ	$\log\beta$	$\mathcal{T}(f_0)$
-0.000577	0.048	0.045	-0.033	0.997
-0.0749	-0.65	-0.602	0.459	0.074
-2.54	-0.282	0.755	0.592	-0.001
-80.3	0.705	-0.256	0.662	-0.001

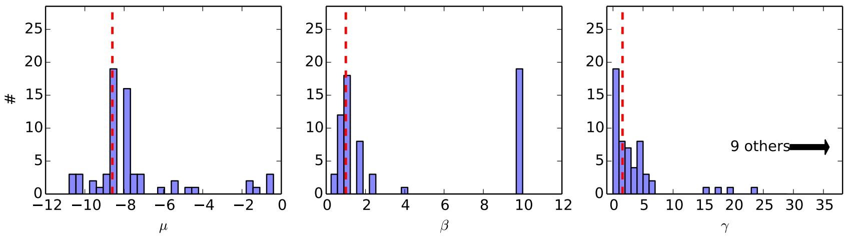

BAS1

λ	$\log\nu$	μ	$\log\beta$	$\mathcal{T}(f_0)$
-0.00334	0.01	0.017	-0.003	1
-0.215	-0.443	-0.886	0.138	0.02
-2.55	0.804	-0.46	-0.377	-0.001
-708	-0.397	0.056	-0.916	0

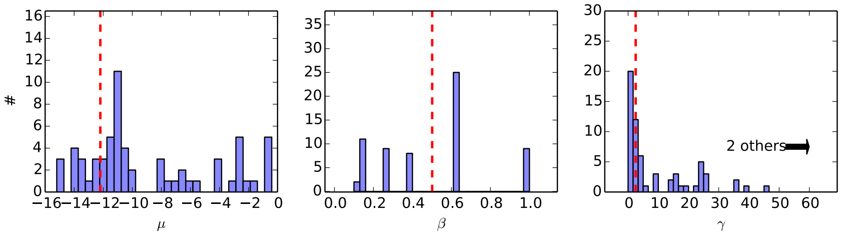

STB5

λ	$\log\nu$	μ	$\log\beta$	$\mathcal{T}(f_0)$
0.000312	-0.221	-0.975	0.014	0.002
-0.014	0.647	-0.145	0.016	0.749
-1.31	0.664	-0.146	0.407	-0.61
-817	-0.304	0.083	0.913	0.259

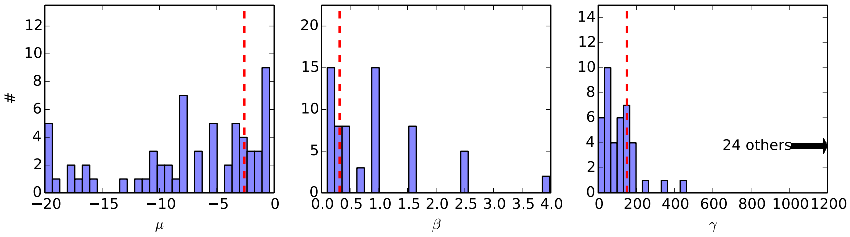

AFT1

λ	$\log\nu$	μ	$\log\beta$	$\mathcal{T}(f_0)$
0.0437	0.447	0.116	-0.887	0.002
-0.000489	-0.001	-0	0.002	1
-3.11	0.842	0.281	0.461	-0
-451	-0.302	0.953	-0.028	0

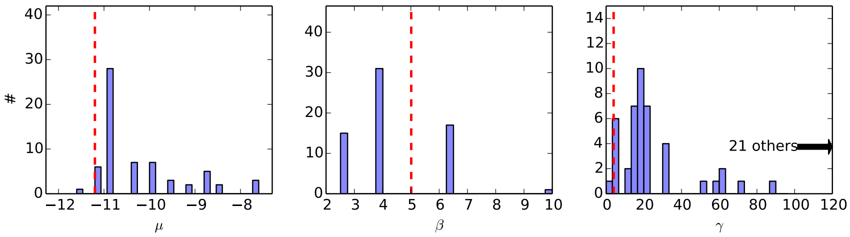

CUP9

λ	$\log\nu$	μ	$\log\beta$	$\mathcal{T}(f_0)$
-0.00113	0.893	0.014	-0	0.45
-0.0189	-0.066	0.993	0.003	0.1
-36.2	0.404	0.107	0.421	-0.805
-8.14e+03	0.187	0.052	-0.907	-0.374

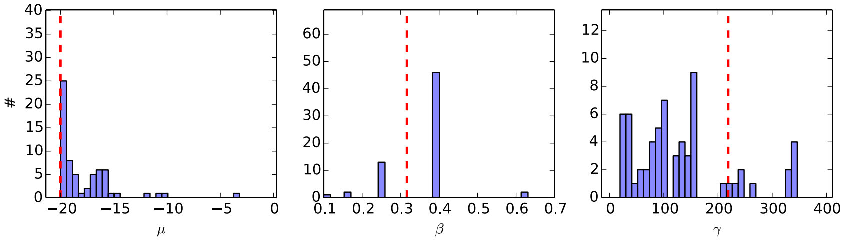

MCM1

λ	$\log\nu$	μ	$\log\beta$	$\mathcal{T}(f_0)$
-0.000392	0.895	-0.005	-0	0.447
-0.00922	-0.036	0.996	0.002	0.084
-26.1	0.421	0.086	0.326	-0.842
-6.13e+03	0.145	0.032	-0.945	-0.29

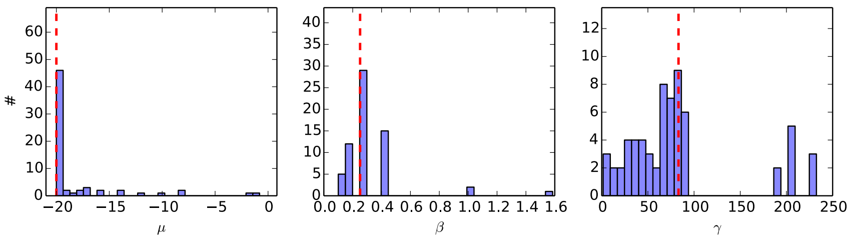

Supplement: Table S3 — Estimates of fitting error. For the 12 TFs in Table 1, we analyze the quality of fit. Columns, from left to right: (A) Eigenvalues and eigenvectors of the Hessian of the likelihood function around the fit maxima. Eigenvectors of the Hessian represent principal directions and the corresponding eigenvalues represent the curvature in those directions, which should be negative at a local maximum. Positive eigenvalues occur if the maximizer did not reach a maximum. Here, the degeneracy represented by is apparent as many fits have an eigenvalue close to zero (flat) or even slightly positive in the direction . For fits subject to the - degeneracy, one can see a second low eigenvalue corresponding to the direction. For computational reasons the Hessian is evaluated using transformed variables , , , and . (B) For each TF, 64 subsets of the full data set were generated by randomly selecting half of the binding sites in the full data set. Maximum likelihood fits were carried out as for the full data set, except that to reduce computation time the grid spacing in the initial four dimensional parameter search was doubled. Shown here are histograms of the resulting parameters. Red dashed lines indicate the maximum likelihood value of each parameter obtained from the full data set. (PDF) [file pcbi.1003683.s003.pdf]
